# The dose**-**response relationship between dairy product intake and all-cause and cardiovascular mortality risk: a systematic review and meta-analysis of prospective cohort studies

**DOI:** 10.3389/fnut.2026.1731841

**Published:** 2026-01-22

**Authors:** Xiaomeng Hu, Kaiyang Wang, Xing Ji, Xinwei Wang, Peiyan Bai, Kailin Huang, Aimei Lu, Jingen Li, Huanlin Wu

**Affiliations:** 1 Dongzhimen Hospital, Beijing University of Chinese Medicine, Beijing, China; 2 Graduate School, Beijing University of Chinese Medicine, Beijing, China

**Keywords:** all-cause mortality, cardiovascular mortality, dairy products, dose-response relationship, meta-analysis of prospective cohort studies

## Abstract

**Background and aims:**

The dose–response relationship between dairy product intake and mortality from all-causes and cardiovascular mortality remains controversial. While dairy products are key sources of essential nutrients, their health effects appear highly heterogeneous, varying by product type, fat content, and processing methods. For example, some studies suggest whole milk increases mortality risk compared to low-fat milk, while other expert reviews from the same year conclude that dairy’s link to CVD risk is neutral regardless of fat content. This systematic review and meta-analysis aimed to clarify this association by examining different dairy categories.

**Methods:**

We systematically searched PubMed, Web of Science, and Embase up to June 19, 2025, for prospective cohort studies in healthy adults that reported risk estimates for the association between dairy intake and all-cause or cardiovascular mortality. Linear and restricted cubic spline models were used for dose–response analysis. From 4,797 retrieved articles, 29 prospective cohort studies involving 1,680,651 participants were included.

**Results and conclusions:**

Our findings indicate that yogurt consumption (200 g/day) was associated with a reduced risk of both all-cause (HR: 0.89; 95% CI: 0.83, 0.96) and cardiovascular mortality (HR: 0.89; 95% CI: 0.83, 0.95). Cheese intake (15 g/day) was linked to a lower risk of CVD mortality only (HR: 0.95; 95% CI: 0.91, 0.99). Milk consumption (200 g/day) was associated with reduced CVD mortality in the non-sex-stratified group (the 7 studies) (HR: 0.86; 95% CI: 0.75, 0.98). Total dairy intake showed a U-shaped association with both mortality outcomes, with an optimal intake of approximately 250–300 g/day. These results highlight that the health effects of dairy are dependent on the specific product type and dose. Fermented dairy products, particularly yogurt, appear to be more beneficial. Our findings do not support the generalized conclusion that all dairy products uniformly protect against mortality, emphasizing the need for more nuanced dietary recommendations.

**Systematic review registration:**

https://www.crd.york.ac.uk/PROSPERO/view/CRD420251067824 Identifier: CRD420251067824.

## 1 Introduction

Cardiovascular diseases (CVD) remain the leading cause of death globally, and identifying modifiable dietary factors that influence CVD and all-cause mortality is a public health priority of the utmost importance. Among the most debated food groups in this context are dairy products. As a staple in many diets worldwide, dairy is a significant source of high-quality protein, calcium, and other essential micronutrients. However, its role in long-term health outcomes is far from clear, with a complex and often contradictory body of evidence contributing to ongoing scientific debate and public confusion (1, 2). The central challenge in understanding the health effects of dairy lies in its heterogeneity. Dairy is not a monolithic food group, and its impact on health is likely dependent on a variety of factors, including the specific product type (e.g., milk, cheese, yogurt), fat content, and processing methods such as fermentation (3, 4). This complexity is reflected in the inconsistent findings from large-scale epidemiological studies. For instance, the Prospective Urban Rural Epidemiology (PURE) study, which included diverse populations from 21 countries, found that higher dairy consumption was associated with a lower risk of both mortality and major cardiovascular events (5). However, other meta-analyses and cohort studies have reported neutral or even adverse associations, particularly with high-fat and non-fermented dairy products like milk and butter (6–8).

A growing body of research suggests that the distinction between fermented and non-fermented dairy products is critical. Fermented dairy, such as yogurt and cheese, has been increasingly linked to more favorable health outcomes. These products contain probiotics, bioactive peptides, and other compounds produced during fermentation that may confer cardiovascular benefits (9, 10). Several meta-analyses have found that consumption of fermented dairy is associated with a reduced risk of CVD and all-cause mortality (11, 12). In contrast, the evidence for non-fermented milk is less consistent, with some studies suggesting a neutral or even increased risk, particularly at high levels of consumption (6, 13). The debate over dairy fat content further complicates the picture. For decades, dietary guidelines have recommended low-fat or non-fat dairy options due to concerns about saturated fat and its effect on LDL cholesterol. However, this paradigm is being challenged by recent evidence. Some studies suggest that the food matrix of dairy may modulate the effects of its saturated fat content, and full-fat dairy products like cheese and yogurt have not been consistently associated with an increased risk of CVD (14–16). In fact, some research indicates that certain fatty acids present in dairy fat may have neutral or even beneficial effects on cardiometabolic health (14).

Given the conflicting findings and the clear heterogeneity in the effects of different dairy products, there is a pressing need for a more nuanced understanding of these relationships. Many existing meta-analyses have focused on total dairy intake or have not adequately distinguished between different product types and their dose–response effects. Therefore, this systematic review and meta-analysis aim to comprehensively evaluate the dose–response relationship between the consumption of specific dairy products—including total dairy, milk, cheese, and yogurt—and the risk of all-cause and cardiovascular mortality.

## 2 Methods

**Figure 1:**
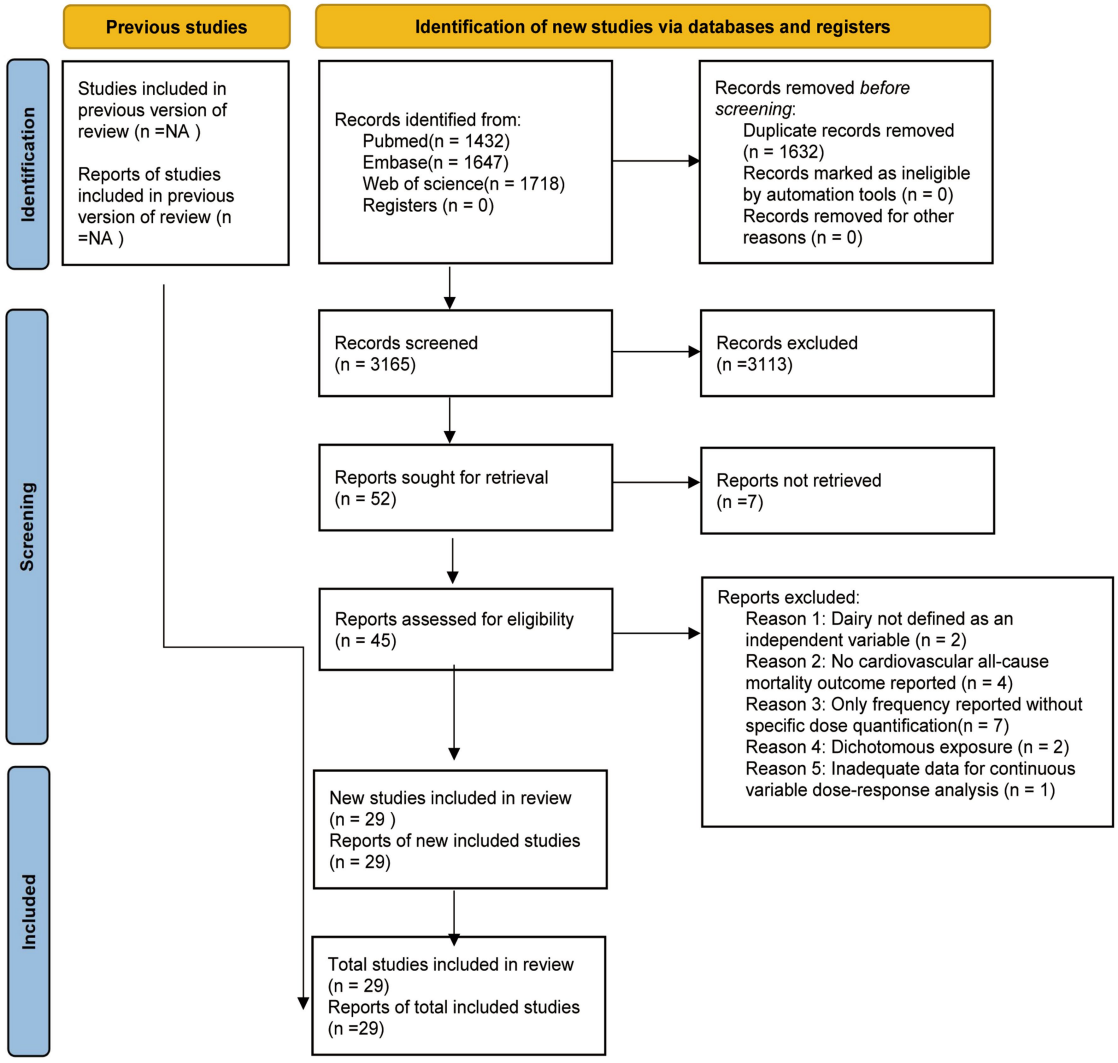
Sorting diagram by exclusion criteria for systematic search of three databases.

This systematic review was registered in the International Prospective Register of Systematic Reviews (PROSPERO) under the registration number CRD420251067824. The review was conducted in accordance with the Meta-analysis of Observational Studies in Epidemiology (MOOSE) guidelines (17). We systematically searched the PubMed, Web of Science, and Embase databases (search cut off date: June 19, 2025). The search strategy included keyword combinations related to dairy product types (e.g., total dairy, milk, cheese, etc.), all-cause mortality, cardiovascular mortality, and prospective cohort studies (see Supplementary Table 1 for detailed search terms). For study selection, prospective cohort study reporting the association between dairy intake and cardiovascular or all-cause mortality were eligible. Details of the criteria were as follows: ① study design: prospective cohort study with a follow-up duration of ≥5 years; ② exposure: dairy product intake (including total dairy, milk, cheese, yogurt, butter, etc.);③ study outcome: cardiovascular mortality or all-cause mortality; ③ Study participants were healthy adults aged ≥18 years, regardless of race or ethnicity, with no history of pre-existing cardiovascular diseases, and those with a history of severe chronic illnesses that may confound mortality outcomes were excluded. ④ study data: provision of risk estimates (including hazard ratio, odds ratio, or relative risk) for the association between dairy exposure and the outcomes of interest, along with uncertainty measures (e.g., 95% confidence intervals) stratified by dairy exposure categories. No time restrictions were applied. Studies were excluded if they met the following criteria: ① not presenting aggregated data; ② were published in non-English; and ③ full-text text were not available even after contacting the authors. After duplicate studies were removed, two researchers independently screened articles retrieved from all three databases by title and abstract to identify potentially eligible studies (see Figure 1 for details). Subsequently, two reviewers independently assessed the full texts of potentially eligible studies in accordance with predefined inclusion and exclusion criteria; disagreements regarding study eligibility during the screening process were resolved through team discussion. For data extraction of study characteristics, 6 researchers were divided into 3 groups (2 persons/group) to independently extract data from included studies. Cross-checks of 15% of the reports were conducted between groups, and discrepancies were arbitrated to ensure extraction accuracy. When analyzing the association between dairy intake and the risk of cardiovascular or all-cause mortality, effect sizes were prioritized from fully adjusted models—specifically, models adjusted for at least body fat, energy intake, and other lifestyle and dietary factors. In addition to study outcomes (cardiovascular mortality, all-cause mortality) and effect sizes (HR values with 95% CIs), the following variables were extracted: ① Study characteristics: study region, follow-up duration, total person-years of observation; ② Participant characteristics: age, sex, ethnicity, total sample size, number of outcome events (cardiovascular deaths, all-cause deaths); ③ Dietary assessment methods: including food frequency questionnaires (FFQ), 24-h dietary recalls, weighed food diaries, etc.; ④ Confounding factor adjustment: including age, body mass index (BMI), energy intake, smoking status, alcohol consumption, physical activity, etc. For missing or unclear information during extraction (e.g., some studies did not specify the validation status of dietary assessment tools or did not directly report total person-years), the following assumptions were made: ① Dietary assessment methods without clear validation were assumed to conform to common standards in the field; ② For studies without direct reporting of person-years, person-years were estimated by ‘total sample size × average follow-up duration’ (used only to assist in judging study scale, not for effect size calculation). For studies with multiple publications, the handling methods were as follows: ① if multiple publications existed for the same cohort, the one with the largest number of cases, most complete data, or longest follow-up duration was prioritized; ② if different types of dairy products were analyzed and reported for the same cohort across separate studies, the cohort could be included multiple times, corresponding to each dairy product category assessed.

### 2.1 Risk of bias and certainty of evidence assessment

Assessment of publication bias was conducted in accordance with the Cochrane Handbook guidelines. Funnel plot visualization, Begg’s test, and Egger’s test were only performed for analyses that included ≥10 studies (i.e., total dairy, cheese, and yogurt) (see Supplementary Figure 1 for details). The Grading of Recommendations Assessment, Development and Evaluation (GRADE) criteria (18, 19) were used to rate the quality of evidence for the associations between each exposure and outcome. The assessment dimensions included: risk of bias within studies, inconsistency between studies, indirectness, imprecision, publication bias, magnitude of effect, and dose–response gradient. All included observational studies were initially rated as high-quality evidence; after assessment using the ROBINS-E tool, the evidence quality was adjusted based on the following criteria (20): ① Downgrading factors: study limitations (risk of bias indicated by ROBINS-E), high heterogeneity (I^2^ > 50% with *p* < 0.10), indirectness (limited generalizability due to characteristics of the population, exposure, or outcome), imprecision (wide 95% CIs or CIs crossing the minimal important threshold of 1.0), and publication bias (evidence of small-study effects); ② Upgrading factors: large effect sizes (HR > 2 or HR < 0.5). The dose–response gradient was not considered an upgrading factor due to the risk of residual confounding (21).

### 2.2 Sensitivity analysis

For all dairy exposure categories, a one-by-one leave-out sensitivity analysis was performed: after sequentially excluding one single study each time, the random-effects model was used to re-pool the effect sizes (HR and 95% CI) of the remaining studies. The impact of each individual study on the robustness of the synthesized results was evaluated by comparing the differences between the re-pooled results and the overall results.

### 2.3 Statistical analysis

We used a random-effects model to calculate pooled hazard ratios (HRs) and their 95% confidence intervals (CIs) for assessing the strength of associations between intake of different dairy products and cardiovascular mortality or all-cause mortality. The standard error of the log HR for each study was obtained via the inverse variance method, which was treated as the estimated variance of the log HR. In the meta-analysis, various risk ratio effect measures (including odds ratio, relative risk, and hazard ratio) were pooled consistently. We also conducted dose–response analyses to separately investigate the associations between dairy intake and two types of mortality outcomes [all-cause mortality and cardiovascular disease (CVD) mortality]. For each dairy category and its corresponding individual outcome (e.g., dairy intake vs. all-cause mortality, dairy intake vs. CVD mortality), dose–response analyses were only performed for the specific “dairy-outcome” pair when there were at least 5 eligible prospective cohort studies reporting that particular outcome. This threshold was set to ensure sufficient statistical power and reliability of the pooled results. Due to insufficient data, dairy categories with fewer than 5 studies available for either mortality outcome (including whole milk, low-fat milk, full-fat yogurt, low-fat yogurt, butter, and others) were not included in the dose–response analyses.

Dose–response analyses were performed using the glst package, and exposure-response relationships were visualized by plotting non-linear and/or linear dose–response curves. The application of cubic spline modeling required the fulfillment of three conditions: ① inclusion of more than 2 quantitative exposure categories (i.e., at least 3); ② availability of the number of cases, non-cases, and person-years of follow-up; ③ reporting of hazard ratios (HR) and its 95% CI. To compare the goodness-of-fit between linear and potential non-linear associations, linear models and restricted cubic spline curves were computed for each study. For studies where the non-linear goodness-of-fit was extremely poor, with abnormal confidence intervals for the predicted non-linear relationships—resulting in the inability to present both linear and non-linear associations of mortality in the same graph (e.g., the association analyses of milk intake with all-cause mortality, cheese intake with CVD mortality, and yogurt intake with CVD mortality)—only the linear associations were retained. All models were individually fitted using the glst procedure in Stata software (22), and then combined via a multivariable random-effects meta-analysis to clarify dose–response trends, which were presented visually in figures. Criteria for determining statistical significance were as follows: ① a pooled HR was considered statistically significant if its 95% CI did not include the null value of 1.00; ② HRs and their 95% CIs were rounded to two decimal places; if the rounded value equaled 1.00, the rounding direction of the original value was indicated (“>“for rounding down, “<“for rounding up); ③ the threshold for statistical significance in all tests (including linear trend tests and heterogeneity tests) was set at *p* < 0.05. Subgroup analysis combined with Q-test and I^2^ statistic was used to identify the sources of heterogeneity; an I^2^ value > 50% indicated the presence of potentially important statistical heterogeneity. In linear model analyses, fixed-effect coefficients and their CIs were extracted from glst outputs using RStudio. Pooled hazard ratio (HR) for the following intake levels were calculated via interpolation to provide standardized reference points: ① 200 g/day for total dairy, milk, and yogurt (this dose fell within the reasonable range of intake measured in all studies and ensured good comparability); ② 15 g/day for cheese. Rules for determining exposure doses were as follows: ① the median or mean value of each quantile category was used as the representative dose; ② for intervals with an open lower bound (e.g., < 50 g/day), the median was set as the midpoint between 0 and the lower bound (e.g., 25 g/day); ③ for closed intervals (e.g., 50–160 g/day), the midpoint of the interval was adopted (e.g., 105 g/day); ④ for intervals with an open upper bound (e.g., > 200 g/day), the midpoint of the highest dose group was set as 1.5 times the lower bound of the interval (e.g., 200 g/day × 1.5 = 300 g/day), in the dose–response meta-analysis curve, the value range of doses must fall between the maximum and minimum doses reported in the original studies, and extrapolation to values outside the fitted dose range is not allowed (22, 23). Standardization of intake units: to ensure comparability across studies that reported intake using different units (e.g., grams, milliliters, servings, cups), we implemented the following hierarchical standardization protocol prior to dose assignment: absolute quantities: intake reported directly in grams (g) or milliliters (mL) was used as provided. Study-defined conversions: For intake reported in units such as “servings” or “cups” where the study explicitly defined the gram/milliliter equivalent, we applied the study-specific conversion factor. Region-based estimation: for the few studies that reported intake in units without a study-specific definition, we estimated the gram equivalent based on the geographical region of the study population and the dietary assessment tool, referencing standard portion sizes commonly used in nutritional epidemiology in that region (e.g., 1 cup = 240 mL in US-based studies). Density Assumption: For liquid dairy intake reported in volume (mL), a density of 1 g/mL was assumed unless stated otherwise, following the method described by Na et al. (24). All conversion factors and their justifications are detailed in Supplementary Table 3.

## 3 Results

A total of 4,797 records were retrieved from the databases, among which 1,632 were duplicates (see Figure 1 for details). Through screening and application of inclusion and exclusion criteria, 45 articles were identified for full-text review. Finally, 29 prospective cohort studies were determined to be included in the final analysis (5, 8, 11, 13, 24–48). These 29 reports covered 29 cohort studies, involving 1,680,651 unique participants. The main characteristics of the selected studies are presented in Supplementary Table 3. Specifically, 3 studies were conducted in China, 1 study across 21 countries on five continents, 3 in Japan, 1 in Italy, 5 in the USA, 3 in The Netherlands, 2 in Australia, 2 in the UK, 1 in Singapore, 3 in Sweden, 1 in Iran, 1 in Russia, 1 in Taiwan (China), 1 in Brazil, and 1 in Denmark. The follow-up duration ranged from 5.8 to 33.0 years, with a mean follow-up duration of 13.4 years for milk intake, 14.6 years for total dairy intake, 15.1 years for cheese intake, and 15.3 years for yogurt intake. Among the 29 studies, 24 used validated food frequency questionnaires (FFQ) to assess dietary intake; the remaining 5 studies employed standardized questionnaires (including 30-day recall of milk type and frequency), 24-h dietary recalls, the Oxford WebQ (a 24-h dietary recall questionnaire), 7-day weighed diet diaries, and 3-day 24-h dietary recalls. All studies that applied multivariable models adjusted for potential confounding factors. Among the included studies, most controlled for key confounders as follows: age (*n* = 28), body mass index (BMI) (*n* = 21), energy intake (*n* = 25), physical activity (*n* = 25), smoking (*n* = 28), alcohol consumption (*n* = 24), and educational level (*n* = 20). Regarding outcome assessment methods, 23 (79.3%) belonged to the category of “official registry/database linkage,” indicating a high reliance on authoritative official data sources in outcome assessment, which enables efficient acquisition of large volumes of outcome information. Three methods (10.3%) were classified as “medical record review”; the relatively low proportion may be attributed to the complexity of obtaining medical records or the limited scenarios in which they are applied. Only one method (3.4%) was “direct follow-up survey,” which accounted for the smallest proportion due to its time-consuming, labor-intensive, and high-cost nature. Two methods (6.9%) were “standardized adjudication procedures”; although their proportion was low, they play a crucial role in ensuring the standardization and accuracy of assessments. Six studies reported results separately for men and women, one cohort included only female participants, and one study presented results for three cohorts individually.

### 3.1 Milk results

#### 3.1.1 Milk-CVD mortality

**Figure 2:**
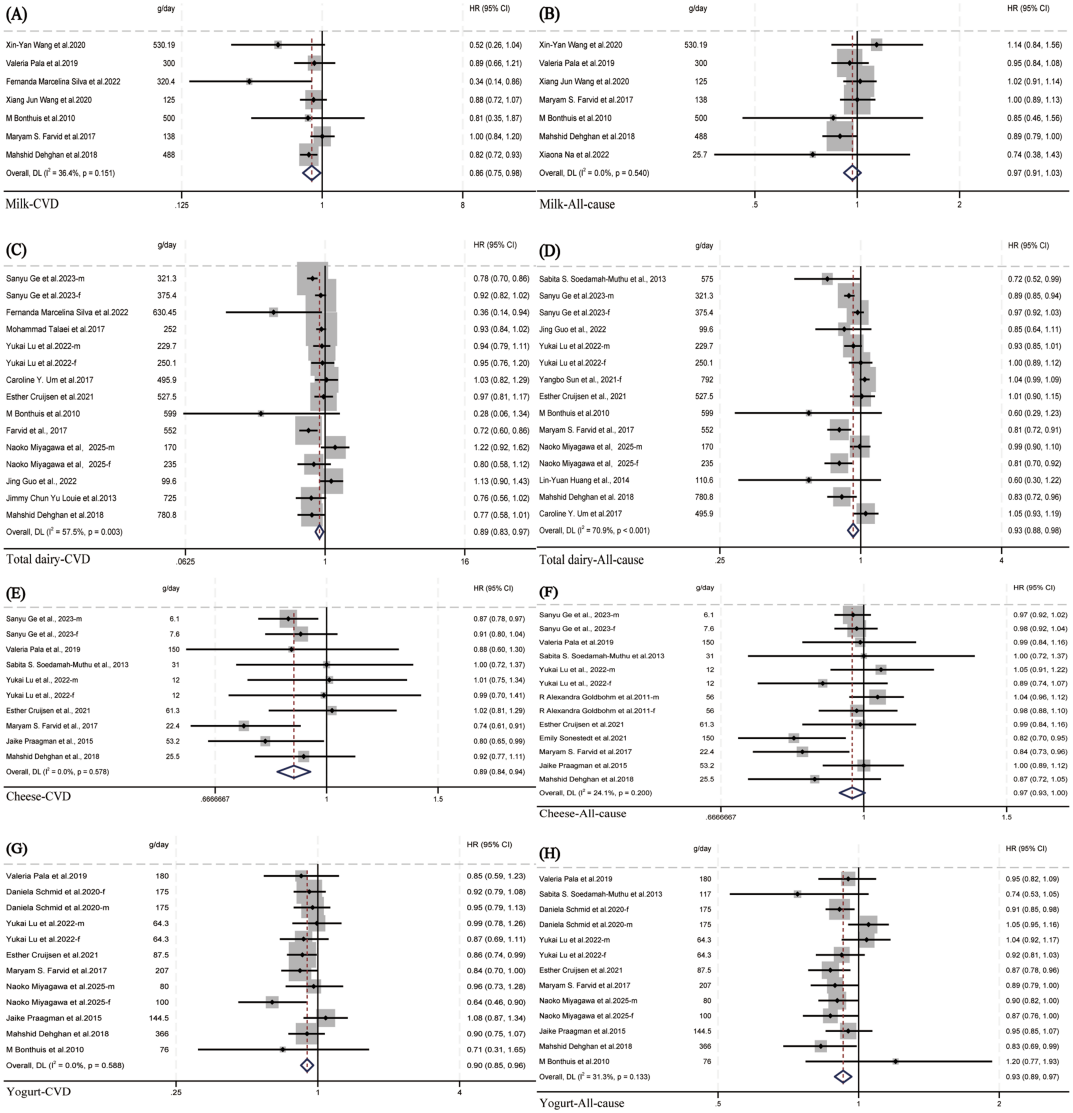
Forest plot of hazard ratios (HRs) for the association between extreme quantiles of dairy product intake and the risk of all-cause and cardiovascular mortality: **(A)**, **(B)** milk; **(C)**, **(D)** total dairy; **(E)**, **(F)** cheese; **(G)**, **(H)** yogurt.

**Figure 3:**
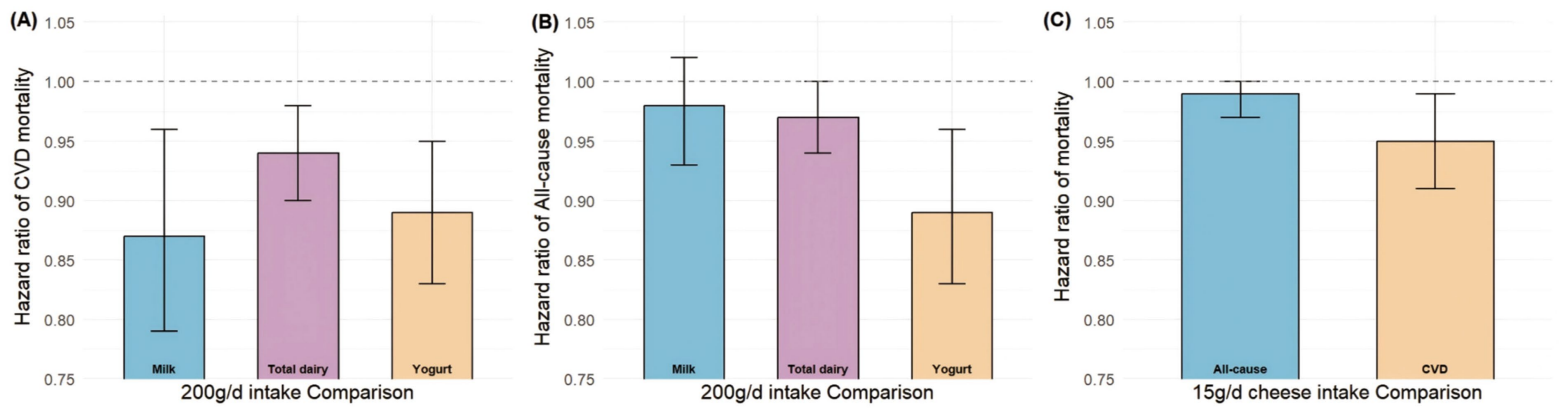
Overall pooled effects of 200 g/d intake of total dairy, milk, and yogurt, and 15 g/d intake of cheese on the risk of all-cause and cardiovascular mortality.

**Figure 4:**
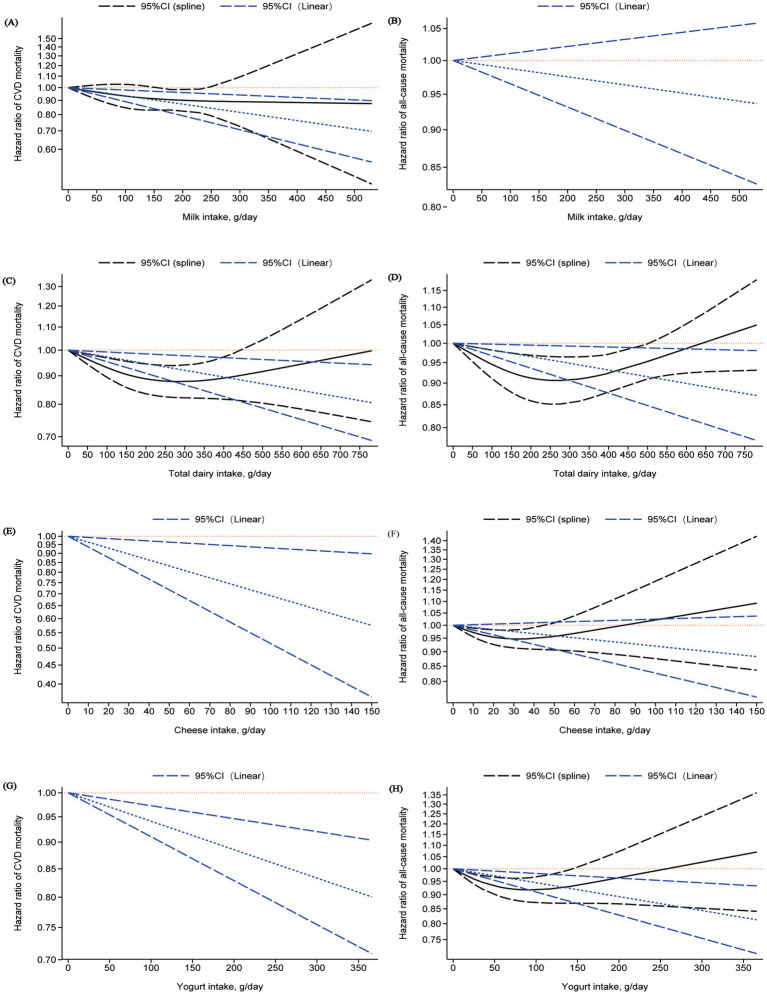
Dose-response relationship between dairy product intake and risk of all-cause and cardiovascular mortality: **(A)**, **(B)** milk; **(C)**, **(D)** total dairy; **(E)**, **(F)** cheese; **(G)**, **(H)** yogurt.

Eleven studies were included to meta-analyze the dose–response relationship between milk intake and incident cardiovascular disease (CVD) mortality risk. When comparing the highest vs. lowest milk intake categories, no association with CVD mortality risk was observed (HR: 0.96; 95% CI: 0.82–1.13; I^2^ = 91.6%; *P*-heterogeneity < 0.001; *n* = 15). Since these 4 studies (which reported sex-specific dose–response relationships) (HR: 1.08; 95% CI: 0.87–1.34; I^2^ = 94.9%; *P*-heterogeneity < 0.001; *n* = 8) were the primary cause of the relatively high overall heterogeneity, we conducted a separate analysis of the remaining 7 studies that did not perform sex-stratified analysis. Following this subgroup analysis of the 7 studies, heterogeneity significantly decreased, with I^2^ dropping from 91.6 to 36.4% (*P*-heterogeneity = 0.151). When re-comparing the highest vs. lowest milk intake categories, a significant association with CVD mortality risk was observed (HR: 0.86; 95% CI: 0.75–0.98; I^2^ = 36.4%; *P*-heterogeneity = 0.151; *n* = 7) (Figure 2). Compared to other dairy product categories, a daily milk intake of 200 g was associated with the greatest reduction in CVD mortality risk (HR: 0.87; 95% CI: 0.79–0.96; moderate certainty) (Figure 3 and Supplementary Table 4). The data were consistent with a linear dose–response relationship (*P*-nonlinearity = 0.670) (Figures 3, 4).

#### 3.1.2 Milk-all-cause mortality

Eleven studies were included to meta-analyze the dose–response relationship between milk intake and incident all-cause mortality risk. When comparing the highest vs. lowest milk intake categories, no association with all-cause mortality risk was observed (HR: 1.02; 95% CI: 0.90–1.16; I^2^ = 96.3%; *P*-heterogeneity < 0.001; *n* = 15). Subgroup analysis revealed that four studies reporting sex-specific (male/female) dose–response relationships were likely the primary source of overall heterogeneity. After excluding these 4 studies and analyzing only the remaining 7 studies that did not stratify by sex, heterogeneity significantly decreased, with I^2^ dropping from 96.3 to 0% (*P*-heterogeneity = 0.540). When re-comparing the highest vs. lowest milk intake categories, no association with all-cause mortality risk was still observed (HR: 0.97; 95% CI: 0.91–1.03; I^2^ = 0%; *P*-heterogeneity = 0.540; *n* = 7) (Figure 2). No association was found between 200 g/day milk intake and all-cause mortality (HR: 0.98; 95% CI: 0.93–1.02; very low certainty) (Figure 3 and Supplementary Table 4), with no evidence of a nonlinear relationship (*P*-nonlinearity = 0.424) (Figures 3, 4).

### 3.2 Total dairy results

#### 3.2.1 Total dairy-CVD mortality

Thirteen studies were included to meta-analyze the dose–response relationship between total dairy intake and incident CVD mortality risk. When comparing the highest vs. lowest total dairy intake categories, a significant association with CVD mortality risk was observed (HR: 0.89; 95% CI: 0.81, 0.97; I^2^ = 62.3%; *P*-heterogeneity < 0.001; *n* = 16). Heterogeneity originated from the study by Huang et al. (47) (HR = 0.1; 95% CI: 0.02–0.52), whose effect size range differed significantly from that of the other included studies and also caused matrix incompatibility during nonlinear dose–response curve fitting and thus compromised the stability and reliability of the overall results. After excluding this study, the pooled effect size was (HR: 0.89; 95% CI: 0.83, 0.97; I^2^ = 57.5%; *P*-heterogeneity = 0.003; *n* = 15) (Figure 2), and the results of the sensitivity analysis for including this outlier study are presented in Supplementary Figure 3. A persistent significant association between total dairy intake and CVD mortality risk was confirmed after exclusion. Compared to other dairy product categories, a daily total dairy intake of 200 g was associated with 6% reduction in the risk of CVD mortality (HR: 0.94; 95% CI: 0.90–0.98; very low certainty) (Figure 3 and Supplementary Table 4) Evidence of a nonlinear dose–response association was detected (*P*-nonlinearity = 0.004): CVD mortality risk decreased with increasing total dairy intake up to ~250–300 g/d, after which risk began to increase (Figures 3, 4).

#### 3.2.2 Total dairy-all-cause mortality

Twelve studies were included to meta-analyze the dose–response relationship between total dairy intake and incident all-cause mortality risk. When comparing the highest vs. lowest total dairy intake categories, a significant association with all-cause mortality risk was observed (HR: 0.93; 95% CI: 0.88, 0.98; I^2^ = 70.9%; *P*-heterogeneity < 0.001; n = 15) (Figure 2). Compared to other dairy product categories, a daily total dairy intake of 200 g was associated with 3% reduction in the risk of all-cause mortality (HR: 0.97; 95% CI: 0.94- < 1.00; very low certainty) (Figure 3 and Supplementary Table 4) Evidence of a nonlinear dose–response association was detected (*P*-nonlinearity = 0.012): All-cause mortality risk decreased with increasing total dairy intake up to ~250–300 g/d, after which risk began to increase (Figures 3, 4).

### 3.3 Cheese results

#### 3.3.1 Cheese-CVD mortality

Eight studies were included to meta-analyze the dose–response relationship between cheese intake and incident CVD mortality risk. When comparing the highest vs. lowest cheese intake categories, a significant association with CVD mortality risk was observed (HR: 0.89; 95% CI: 0.84, 0.94; I^2^ = 0%; *P*-heterogeneity = 0.578; *n* = 10) (Figure 2). A daily cheese intake of 15 g was associated with a 5% reduction in CVD mortality risk (HR: 0.95; 95% CI: 0.91, 0.99; moderate certainty) (Figure 3 and Supplementary Table 4). The data were consistent with a linear dose–response relationship (*P*-nonlinearity = 0.077) (Figures 3, 4).

#### 3.3.2 Cheese-all-cause mortality

Ten studies were included to meta-analyze the dose–response relationship between cheese intake and incident all-cause mortality risk. When comparing the highest vs. lowest cheese intake categories, no association with all-cause mortality risk was observed (HR: 0.97; 95% CI: 0.93, 1.00; I^2^ = 24.1%; *P*-heterogeneity = 0.200; *n* = 13) (Figure 2). No association was found between 15 g/day cheese intake and all-cause mortality (HR: 0.99; 95% CI: 0.97, 1.00; very low certainty) (Figure 3 and Supplementary Table 4) Evidence of a nonlinear dose–response association was detected (*P*-nonlinearity = 0.017): All-cause mortality risk decreased with increasing cheese intake up to ~30–35 g/d, after which risk began to increase (Figures 3, 4).

### 3.4 Yogurt results

#### 3.4.1 Yogurt-CVD mortality

Nine studies were included to meta-analyze the dose–response relationship between yogurt intake and incident CVD mortality risk. When comparing the highest vs. lowest yogurt intake categories, a significant association with CVD mortality risk was observed (HR: 0.90; 95% CI: 0.85, 0.96; I^2^ = 0%; *P*-heterogeneity = 0.588; *n* = 12) (Figure 2). A daily yogurt intake of 200 g was associated with a 11% reduction in CVD mortality risk (HR: 0.89; 95% CI: 0.83, 0.95; moderate certainty) (Figure 3 and Supplementary Table 4). The data were consistent with a linear dose–response relationship (*P*-nonlinearity = 0.158) (Figures 3, 4).

#### 3.4.2 Yogurt-all-cause mortality

Ten studies were included to meta-analyze the dose–response relationship between yogurt intake and incident all-cause mortality risk. When comparing the highest vs. lowest yogurt intake categories, a significant association with all-cause mortality risk was observed (HR: 0.93; 95% CI: 0.89, 0.97; I^2^ = 31.3%; *P*-heterogeneity = 0.133; *n* = 13) (Figure 2). A daily yogurt intake of 200 g was associated with a 11% reduction in all-cause mortality risk (HR: 0.89; 95% CI: 0.83, 0.96; moderate certainty) (Figure 3 and Supplementary Table 4). Evidence of a nonlinear dose–response association was detected (*P*-nonlinearity = 0.001): All-cause mortality risk decreased with increasing yogurt intake up to ~75–100 g/d, after which risk began to increase (Figures 3, 4).

### 3.5 Risk of bias

Owing to the non - random observational nature of the studies, the likelihood of residual confounding, and the likelihood of measurement error in dietary assessment, no study was judged to have a low risk of bias. All publications were judged to have a moderate risk of bias. Specifically, Supplementary Table 2 lists the ROBINS - E descriptions and decision criteria for each risk of bias domain. The results of the risk of bias were considered as part of the GRADE assessment (for complete details of the GRADE evidence, please refer to Supplementary Table 4).

### 3.6 Heterogeneity analysis

To explore potential sources of heterogeneity, subgroup analyses and univariate/multivariate meta-regressions were conducted for key associations: For the association between milk intake and CVD mortality, subgroup analyses were stratified by sex (non-sex-stratified group, female, male), region (Asia vs. Europe and America), total sample size (<10,000 vs. ≥10,000), and follow-up duration (<10 years vs. ≥10 years). As reflected in Supplementary Tables 5.1, 5.2, sex (*p* = 0.058) showed marginally significant associations with between-study heterogeneity, while subgroup analyses based on all other factors failed to reduce the overall heterogeneity (see Supplementary Figure 4), with limited explanatory power for heterogeneity observed for these factors.

For the association between total dairy intake and all-cause mortality, subgroup analyses were stratified by the same factors. As shown in Supplementary Tables 5.3, 5.4, none of these factors (all *p* > 0.10) showed significant or marginally significant associations with between-study heterogeneity, and these subgroup analyses did not reduce the heterogeneity (see Supplementary Figure 4). Thus, the exclusion strategy (validated by sensitivity analysis) was a more feasible solution to improve model stability.

### 3.7 Sensitivity analysis and publication bias assessment

For all dairy exposure categories, removing any single study at a time did not alter the significance or direction of the association (Supplementary Figure 2).

Visual inspection of the funnel plot and formal statistical tests revealed no evidence of publication bias in the studies investigating total dairy, cheese, or yogurt. For total dairy and CVD mortality, the Begg’s test (Kendall’s tau = −0.083, *p* = 0.520) and Egger’s test (*t* = −0.52, df = 13, *p* = 0.610) were non-significant. When the standard error approached 0, the limiting estimate was −0.435 (95% CI: −2.234, 1.364). For total dairy and all-cause mortality, the Begg’s test (Kendall’s tau = −0.073, *p* = 0.586) and Egger’s test (*t* = −1.83, df = 13, *p* = 0.090) showed no significance. The limiting estimate was −1.491 (95% CI: −3.250, 0.267) when the standard error neared 0. Regarding cheese and all-cause mortality, non-significant results were observed in the Begg’s test (Kendall’s tau = −0.218, *p* = 0.300) and Egger’s test (*t* = −1.17, df = 11, *p* = 0.268). The limiting estimate was −0.793 (95% CI: −2.290, 0.704) at a near-zero standard error. Similarly, for yogurt and all-cause mortality, the Begg’s test (Kendall’s tau = −0.128, *p* = 0.542) and Egger’s test (*t* = −0.20, df = 11, *p* = 0.848) were non-significant, with a limiting estimate of −0.194 (95% CI: −2.366, 1.979) when the standard error approached 0. These findings indicate no significant asymmetry in the funnel plots, suggesting the absence of publication bias in the studies focusing on these three dairy categories. To further verify the robustness of the pooled results and address the impact of potential outliers, a targeted sensitivity assessment was additionally performed for the study by Huang et al. (47), as detailed below: this study was excluded due to its extreme outlier effect size (HR = 0.1; 95% CI: 0.02–0.52). A retrospective review of the original paper revealed that the significant association between total dairy intake and overall cardiovascular disease (CVD) mortality reported in this study stemmed from its extreme protective effect on stroke mortality, which reflected the unique characteristics or local mechanisms of the study population rather than a universal effect of dairy products. Moreover, this study caused matrix incompatibility during nonlinear dose–response curve fitting, which disrupted model convergence. The results of the special sensitivity analysis showed that the pooled effect size remained consistent before and after the exclusion of this study (HR = 0.89 in both cases), the heterogeneity decreased moderately (I^2^ decreased from 62.3 to 57.5%), and the stable fitting of the nonlinear dose–response relationship was only achievable after the exclusion.

## 4 Discussion

This systematic review and meta-analysis significantly advances our understanding of the complex relationship between dairy consumption and mortality by moving beyond generalized assessments of total dairy intake. The most significant innovation of our work lies in the detailed, product-specific, dose–response analysis, which uncovers critical nuances previously obscured by heterogeneity. We demonstrate that the health effects of dairy are not monolithic but are distinctly modulated by product type (fermented vs. non-fermented), dose, the specific mortality outcome, and even the consumer’s sex. Our findings collectively argue for a paradigm shift in dietary recommendations, moving away from broad advice on dairy towards more precise, evidence-based guidance.

### 4.1 The striking consistency of fermented dairy’s protective effects

A cornerstone finding of this analysis is the robust and remarkably consistent protective effect of fermented dairy products, particularly yogurt and cheese, against cardiovascular mortality. For both yogurt and cheese, the association with reduced CVD mortality was characterized by zero statistical heterogeneity (I^2^ = 0%). This is a profound and novel observation, suggesting that the cardiovascular benefits of these foods are stable and generalizable across diverse populations and study designs. This consistency strongly implies that the underlying biological mechanisms are potent and not easily swayed by confounding factors. As a representative fermented dairy product, yogurt demonstrates robust, high-quality evidence for reducing mortality risk, making it a priority recommendation in dietary guidelines (39, 49, 50). Recent research corroborates the unique benefits of fermentation, which creates bioactive peptides, vitamin K2 (menaquinones), and probiotics that are not present in non-fermented milk (3, 51). These compounds are known to improve cardiometabolic health by modulating gut microbiota, reducing inflammation, improving insulin sensitivity, and regulating blood pressure (52, 53). Our analysis provides strong, pooled quantitative evidence to support the hypothesis that the food matrix of fermented dairy confers benefits that transcend its basic nutrient profile.

### 4.2 The role of dairy polar lipids in relation to CVD

Beyond the well-established benefits of fermentation-derived bioactive compounds, emerging evidence highlights the critical role of dairy polar lipids (PL) in relation to CVD—specifically in mediating cardiovascular protective effects by inhibiting platelet aggregation, alleviating chronic inflammation, and improving lipid metabolism. This finding directly addresses the misconception that dairy fat is pro-inflammatory. As emphasized, dairy PL (predominantly localized in the milk fat globule membrane, MFGM) are key bioactive components that exert anti-CVD effects through these three interconnected mechanisms (54). Recent human and preclinical studies provide robust support for these interconnected mechanisms, as detailed below:

Antiplatelet and antithrombotic effects: Lordan et al. (55) confirmed that dairy-derived polar lipids (PLs), especially phosphatidylethanolamine (PE), sphingomyelin (SM), and phosphatidylcholine (PC) enriched after fermentation, possess significant antiplatelet activity. These PLs can inhibit platelet aggregation induced by platelet-activating factor (PAF) and thrombin. Among them, the most bioactive yogurt (fermented by *Streptococcus thermophilus* and *Lactobacillus acidophilus*) exhibited an IC₅₀ value as low as 14.7 ± 6.0 μg against thrombin-induced platelet aggregation. Additionally, molecular species such as PC(18:0/18:1), PE(18:1/18:2), and SM(d18:0/22:0) showed a significant negative correlation with antiplatelet activity. The study also found that PLs with similar anti-PAF activity exist in fermented caprine milk cheese, further verifying the conserved antithrombotic function of PLs across different milk sources (55).

Regulation of lipid metabolism and reduction of cholesterol absorption: Vors et al. (56) conducted two pivotal trials on milk PL (rich in sphingomyelin, SM): a 4-week double-blind randomized controlled trial (RCT) with 58 overweight postmenopausal women showed that daily supplementation with 3 g or 5 g milk PL significantly reduced fasting and postprandial cholesterol levels, as well as CVD-related lipid markers (e.g., pro-atherogenic lipid species). The 5 g dose was more effective, increasing faecal coprostanol excretion and decreasing intestinal chylomicron formation—without disrupting gut microbiota or short-chain fatty acid profiles. A subsequent crossover trial in ileostomy patients further confirmed that milk PL reduces cholesterol absorption and enhances ileal cholesterol efflux via SM-cholesterol co-excretion, providing the direct human evidence for its role in improving cardiometabolic health (56).

Systematic anti-inflammatory and lipid metabolism-regulating effects on cardiovascular protection: Venkat et al. (57) confirmed through a systematic review of 378 studies that milk-derived polar lipids [including glycerophospholipids such as phosphatidylcholine (PC) and phosphatidylethanolamine (PE), as well as sphingolipids such as sphingomyelin (SM)] exert stable cardiovascular disease-resistant effects. The core mechanisms include reducing low-density lipoprotein cholesterol (LDL-C), total cholesterol, and atherogenic sphingomyelin/ceramide (Cer) species. Additionally, they regulate gut-specific interactions (e.g., sphingomyelin-cholesterol co-excretion) and lipid metabolism, alleviate inflammatory responses, without disrupting intestinal homeostasis (57).

Species-conserved anti-inflammatory mechanisms of core PL subclasses: Chen et al. (58) used lipidomics (ultra-high-performance liquid chromatography-triple quadrupole linear ion trap mass spectrometry, UHPLC-QTRAP-MS) to compare bovine milk (BM) and donkey milk (DM) PL profiles, identifying that core PL subclasses (SM, PC, PE) in both milks exhibit clear anti-CVD potential. Specifically, SM regulates lipid metabolism to improve lipid profiles, while PC and PE alleviate chronic inflammation by modulating macrophage polarization and inhibiting the NF-κB inflammatory pathway—a conserved mechanism that underscores the CVD-protective value of dairy PL across mammalian milk sources (58).

In conclusion, these recent studies demonstrate that dairy PL are key mediators of dairy’s cardiovascular benefits, with their antiplatelet, anti-inflammatory, and lipid-regulating effects acting synergistically to counter the wrong characterization of dairy fat as pro-inflammatory. This highlights the value of dairy PL as natural bioactive ingredients for CVD prevention.

### 4.3 Unveiling non-linearity: the importance of dose and moderation

Another key innovation of our study is the identification of non-linear dose–response relationships for several dairy categories. For total dairy intake, we identified a significant U-shaped association for both all-cause and CVD mortality, with an optimal intake around 250–300 g/day. This finding challenges a simplistic “more is better” or “less is better” approach, providing a quantitative basis for the principle of moderation. The initial protective trend at lower doses is likely due to the beneficial effects of calcium, protein, and other nutrients. Critically, the subsequent increase in risk at higher intakes may not be attributable to saturated fat alone but could reflect a shift in the composition of dairy consumption at these levels, potentially characterized by a higher proportion of non-fermented, high-fat products such as butter and cream. This interpretation aligns with the fact that the adverse metabolic consequences of excessive calorie and saturated fat intake may eventually overwhelm the initial benefits when derived from specific, less beneficial dairy sources (4). Importantly, the shape of the dose–response curve for “total dairy” appears to be significantly modulated by the specific product mix within an individual’s diet. Our finding of a U-shaped curve for cheese and all-cause mortality is also consistent with a recent 2023 meta-analysis, placing our results at the forefront of current research (59). This presents a compelling paradox: cheese is itself high in saturated fat. Its distinct dose–response relationship underscores that the health impact of a dairy product is not defined solely by its nutrient profile but is profoundly shaped by its food matrix and processing methods, such as fermentation. The fermentation process and the complex physical structure of cheese may modify the metabolic effects of its saturated fat and confer additional benefits through bioactive peptides and probiotics. Similarly, the “reverse U-shaped” curve for yogurt and all-cause mortality, with a nadir at 75–100 g/day, suggests a point of diminishing returns. This nonlinear characteristic is also supported by other studies: one study found a non-linear association between yogurt intake and the risks of all-cause mortality and cardiovascular disease mortality, where the risk no longer decreases further when daily yogurt intake exceeds 0.5 servings (approximately 122 grams) (50). While yogurt remained protective at 200 g/day, the optimal benefit was achieved at a more moderate dose. Collectively, these nuanced, non-linear relationships highlight that a one-size-fits-all recommendation for dairy intake is inadequate. The level of detail provided by our models offers a data-driven foundation for refining public health guidelines. Future recommendations should move beyond aggregate intake or single nutrients and towards providing nuanced guidance that considers specific dairy products, their fermentation status, and optimal consumption ranges to maximize population health benefits.

### 4.4 Sex-stratified analysis of milk: heterogeneity sources and core findings

Four studies with sex-stratified(HR: 1.08; 95% CI: 0.87–1.34; I^2^ = 94.9%; P-heterogeneity < 0.001; *n* = 8) reports were the primary cause of high heterogeneity in the initial analysis. Excluding these, analysis of seven non-sex-stratified studies (HR: 0.86; 95% CI: 0.75–0.98; I^2^ = 36.4%; P-heterogeneity = 0.151; *n* = 7) showed significantly reduced heterogeneity, confirming milk intake was associated with lower cardiovascular disease (CVD) mortality, with no significant sex-specific differences. Uppsala University’s 2024 study indicated that milk intake is associated with an increased risk of cardiovascular disease (CVD) in women (60). Due to the limitation of the number of studies, our study did not find a clear dose–response relationship between milk intake and male/female subgroups. These findings suggest that the sex-moderating effect on the association between milk intake and CVD mortality may depend on study design or population characteristics, requiring further validation. Our study clarifies that including sex-stratified studies is a key heterogeneity source in such meta-analyses and confirms milk’s cardiovascular protective effect in the general population. It also highlights that future sex-effect research should uniformly incorporate complete sex-stratified data to avoid heterogeneity confounding core conclusions.

### 4.5 Strengths and limitations

The primary strength and innovation of this meta-analysis is the rigorous, product-specific dose–response analysis, which allowed for the identification of non-linear relationships and optimal intake levels. The large sample size, inclusion of recent prospective cohort studies, and the robust, zero-heterogeneity findings for fermented dairy and CVD mortality are other major strengths.

However, our study has limitations. First, the included studies are observational, which precludes causal inference. Second, despite our detailed analysis, the evidence certainty for some outcomes, particularly for total dairy intake, was rated as “very low” due to residual heterogeneity and potential for confounding. Third, dietary data were based on self-report, which is subject to measurement error. Furthermore, the analytical depth of this synthesis was constrained by the reporting granularity in the original studies. For example, the number of studies reporting intake for specific dairy subcategories (such as whole milk versus low-fat milk) was limited (fewer than five), precluding meaningful stratified analyses and hindering the investigation of the independent health effects of products with different fat contents. Finally, most included studies were from North America and Europe, which may limit the generalizability of our findings to other populations with different dietary patterns and genetic backgrounds.

## Data Availability

The original contributions presented in the study are included in the article/Supplementary material, further inquiries can be directed to the corresponding authors.

## References

[1] Zhuang P Liu X Li Y Ao Y Wu Y Ye H . A global analysis of dairy consumption and incident cardiovascular disease. Nat Commun. (2025) 16:437. doi: 10.1038/s41467-024-55585-0, 39762253 PMC11704150

[2] Lamarche B Astrup A Eckel RH Feeney E Givens I Krauss RM . Regular-fat and low-fat dairy foods and cardiovascular diseases: perspectives for future dietary recommendations. Am J Clin Nutr. (2025) 121:956–64. doi: 10.1016/j.ajcnut.2025.03.009, 40088974 PMC12107492

[3] Astrup A Geiker NRW Magkos F. Effects of full-fat and fermented dairy products on cardiometabolic disease: food is more than the sum of its parts. Adv Nutr. (2019) 10:924S–30S. doi: 10.1093/advances/nmz069, 31518411 PMC6743821

[4] Guo J Astrup A Lovegrove JA Gijsbers L Givens DI Soedamah-Muthu SS. Milk and dairy consumption and risk of cardiovascular diseases and all-cause mortality: dose-response meta-analysis of prospective cohort studies. Eur J Epidemiol. (2017) 32:269–87. doi: 10.1007/s10654-017-0243-1, 28374228 PMC5437143

[5] Dehghan M Mente A Rangarajan S Sheridan P Mohan V Iqbal R . Association of dairy intake with cardiovascular disease and mortality in 21 countries from five continents (PURE): a prospective cohort study. Lancet. (2018) 392:2288–97. doi: 10.1016/S0140-6736(18)31812-9, 30217460

[6] Larsson SC Crippa A Orsini N Wolk A Michaëlsson K. Milk consumption and mortality from all causes, cardiovascular disease, and cancer: a systematic review and meta-analysis. Nutrients. (2015) 7:7749–63. doi: 10.3390/nu7095363, 26378576 PMC4586558

[7] Arnesen EK Christensen JJ Laake I Carlsen MH Veierød MB Retterstøl K. Low-fat and whole milk consumption in relation to cardiovascular disease-related and all-cause mortality: a prospective cohort study in 3 Norwegian counties. Am J Clin Nutr. (2025) 122:1075–85. doi: 10.1016/j.ajcnut.2025.07.035, 40759395 PMC12674078

[8] Zhang Y Chadaideh KS Li Y Li Y Gu X Liu Y . Butter and plant-based oils intake and mortality. JAMA Intern Med. (2025) 185:549–60. doi: 10.1001/jamainternmed.2025.0205, 40048719 PMC11886867

[9] Zhang K Chen X Zhang L Deng Z. Fermented dairy foods intake and risk of cardiovascular diseases: a meta-analysis of cohort studies. Crit Rev Food Sci Nutr. (2020) 60:1189–94. doi: 10.1080/10408398.2018.1564019, 30652490

[10] Tapsell LC. Fermented dairy food and CVD risk. Br J Nutr. (2015) 113:S131–5. doi: 10.1017/S0007114514002359, 26148916

[11] Farvid MS Malekshah AF Pourshams A Poustchi H Sepanlou SG Sharafkhah M . Dairy food intake and all-cause, cardiovascular disease, and cancer mortality: the Golestan cohort study. Am J Epidemiol. (2017) 185:697–711. doi: 10.1093/aje/kww139, 28369205 PMC5860026

[12] Akyil S Winkler S Meyer D Kiesswetter E Kussmann M Schwingshackl L . Association between dairy intake and multiple health outcomes: a scoping review of systematic reviews and meta-analyses. Eur J Clin Nutr. (2025) 80. doi: 10.1038/s41430-025-01639-5, 40715472 PMC12783052

[13] Michaëlsson K Wolk A Langenskiöld S Basu S Warensjo Lemming E Melhus H . Milk intake and risk of mortality and fractures in women and men: cohort studies. BMJ. (2014) 349:g6015. doi: 10.1136/bmj.g6015, 25352269 PMC4212225

[14] Trieu K Bhat S Dai Z Leander K Gigante B Qian F . Biomarkers of dairy fat intake, incident cardiovascular disease, and all-cause mortality: a cohort study, systematic review, and meta-analysis. PLoS Med. (2021) 18:e1003763. doi: 10.1371/journal.pmed.1003763, 34547017 PMC8454979

[15] Ding M Li J Qi L Ellervik C Zhang X Manson JE . Associations of dairy intake with risk of mortality in women and men: three prospective cohort studies. BMJ. (2019) 367:l6204. doi: 10.1136/bmj.l6204 31776125 PMC6880246

[16] Astrup A Magkos F Bier DM Brenna JT de Oliveira Otto MC Hill JO . Saturated fats and health: a reassessment and proposal for food-based recommendations: JACC state-of-the-art review. J Am Coll Cardiol. (2020) 76:844–57. doi: 10.1016/j.jacc.2020.05.077, 32562735

[17] Stroup DF Berlin JA Morton SC Olkin I Williamson GD Rennie D . Meta-analysis of observational studies in epidemiology: a proposal for reporting. Meta-analysis of observational studies in epidemiology (MOOSE) group. JAMA. (2000) 283:2008–12. doi: 10.1001/jama.283.15.2008, 10789670

[18] Guyatt G Oxman AD Akl EA Kunz R Vist G Brozek J . GRADE guidelines: 1. Introduction-GRADE evidence profiles and summary of findings tables. J Clin Epidemiol. (2011) 64:383–94. doi: 10.1016/j.jclinepi.2010.04.026, 21195583

[19] Schünemann H. Brożek J. Guyatt G. Oxman A. (Eds). GRADE handbook for grading quality of evidence and strength of recommendations. GRADE Working Group. (2013). Available online at: https://gdt.gradepro.org/app/handbook/handbook.html (Accessed September 30, 2025).

[20] Schünemann HJ Cuello C Akl EA Mustafa RA Meerpohl JJ Thayer K . GRADE guidelines: 18. How ROBINS-I and other tools to assess risk of bias in nonrandomized studies should be used to rate the certainty of a body of evidence. J Clin Epidemiol. (2019) 111:105–14. doi: 10.1016/j.jclinepi.2018.01.012, 29432858 PMC6692166

[21] Murad MH Verbeek J Schwingshackl L Filippini T Vinceti M Akl EA . GRADE GUIDANCE 38: updated guidance for rating up certainty of evidence due to a dose-response gradient. J Clin Epidemiol. (2023) 164:45–53. doi: 10.1016/j.jclinepi.2023.09.011, 37777140

[22] Luo M Lin X Liu R Liu H Chen T Hu Q . Implementation of dose-response meta-analysis in Stata software. Evid Based Med. (2014) 14:182–7.

[23] Mousavi SM Zargarzadeh N Rigi S Persad E Pizarro AB Hasani-Ranjbar S . Egg consumption and risk of all-cause and cause-specific mortality: a systematic review and dose-response meta-analysis of prospective studies. Adv Nutr. (2022) 13:1762–73. doi: 10.1093/advances/nmac040, 35396834 PMC9526855

[24] Na X Lan H Wang Y Tan Y Zhang J Zhao A. Association between milk intake and all-cause mortality among Chinese adults: a prospective study. Nutrients. (2022) 14:292. doi: 10.3390/nu14020292, 35057475 PMC8779580

[25] Lu Y Sugawara Y Matsuyama S Fukao A Tsuji I. Association of dairy intake with all-cause, cancer, and cardiovascular disease mortality in Japanese adults: a 25-year population-based cohort. Eur J Nutr. (2022) 61:1285–97. doi: 10.1007/s00394-021-02734-6, 34750640 PMC8921048

[26] Bonthuis M Hughes MCB Ibiebele TI Green AC van der Pols JC. Dairy consumption and patterns of mortality of Australian adults. Eur J Clin Nutr. (2010) 64:569–77. doi: 10.1038/ejcn.2010.45, 20372173

[27] Michaëlsson K Byberg L. Mixing of apples and oranges in milk research: a cohort analysis of non-fermented milk intake and all-cause mortality. Nutrients. (2020) 12:1393. doi: 10.3390/nu12051393, 32413977 PMC7284719

[28] Sonestedt E Borné Y Wirfält E Ericson U. Dairy consumption, lactase persistence, and mortality risk in a cohort from southern Sweden. Front Nutr. (2021) 8:779034. doi: 10.3389/fnut.2021.779034, 34901125 PMC8652079

[29] Goldbohm RA Chorus AMJ Galindo Garre F Schouten LJ van den Brandt PA. Dairy consumption and 10-y total and cardiovascular mortality: a prospective cohort study in the Netherlands. Am J Clin Nutr. (2011) 93:615–27. doi: 10.3945/ajcn.110.000430, 21270377

[30] Wang XY Liu FC Yang XL Li JX Cao J Lu XF . Association of cardiovascular diseases with milk intake among general Chinese adults. Chin Med J. (2020) 133:1144–54. doi: 10.1097/CM9.0000000000000786, 32433046 PMC7249710

[31] Ge S Zha L Sobue T Kitamura T Iso H Ishihara J . Associations between dairy intake and mortality due to all-cause and cardiovascular disease: the Japan public health Center-based prospective study. Eur J Nutr. (2023) 62:2087–104. doi: 10.1007/s00394-023-03116-w, 36943492 PMC10349701

[32] Pala V Sieri S Chiodini P Masala G Palli D Mattiello A . Associations of dairy product consumption with mortality in the European prospective investigation into Cancer and nutrition (EPIC)-Italy cohort. Am J Clin Nutr. (2019) 110:1220–30. doi: 10.1093/ajcn/nqz183, 31435641

[33] Soedamah-Muthu SS Masset G Verberne L Geleijnse JM Brunner EJ. Consumption of dairy products and associations with incident diabetes, CHD and mortality in the Whitehall II study. Br J Nutr. (2013) 109:718–26. doi: 10.1017/S0007114512001845, 22676797

[34] Louie JCY Flood VM Burlutsky G Rangan AM Gill TP Mitchell P. Dairy consumption and the risk of 15-year cardiovascular disease mortality in a cohort of older Australians. Nutrients. (2013) 5:441–54. doi: 10.3390/nu5020441, 23389303 PMC3635204

[35] Silva FM Giatti L Diniz M d FHS Brant LCC Barreto SM. Dairy product consumption reduces cardiovascular mortality: results after 8 year follow-up of ELSA-brasil. Eur J Nutr. (2022) 61:859–69. doi: 10.1007/s00394-021-02686-x, 34626206

[36] Wang S Liu Y Cai H Li Y Zhang X Liu J . Decreased risk of all-cause and heart-specific mortality is associated with low-fat or skimmed milk consumption compared with whole milk intake: a cohort study. Clin Nutr. (2021) 40:5568–75. doi: 10.1016/j.clnu.2021.09.012, 34656953

[37] Lin Z Zeng M Sui Z Wu Y Tang X Liu T. Moderate full-fat and low-fat yoghurt consumption correlates with reduced mortality risk: a large-scale prospective analysis. J Glob Health. (2025) 15:4014. doi: 10.7189/jogh.15.04014, 39820103 PMC11737816

[38] Talaei M Koh WP Yuan JM Pan A. The association between dairy product intake and cardiovascular disease mortality in Chinese adults. Eur J Nutr. (2017) 56:2343–52. doi: 10.1007/s00394-016-1274-1 27447793

[39] Schmid D Song M Zhang X Willett WC Vaidya R Giovannucci EL . Yogurt consumption in relation to mortality from cardiovascular disease, cancer, and all causes: a prospective investigation in 2 cohorts of US women and men. Am J Clin Nutr. (2020) 111:689–97. doi: 10.1093/ajcn/nqz345, 31968071 PMC7049530

[40] Guo J Givens DI Heitmann BL. Association between dairy consumption and cardiovascular disease events, bone fracture and all-cause mortality. PLoS One. (2022) 17:e0271168. doi: 10.1371/journal.pone.0271168, 36083880 PMC9462570

[41] Wang XJ Jiang CQ Zhang WS Zhu F Jin YL Woo J . Milk consumption and risk of mortality from all-cause, cardiovascular disease and cancer in older people. Clin Nutr. (2020) 39:3442–51. doi: 10.1016/j.clnu.2020.03.003, 32229169

[42] Sun Y Liu B Snetselaar LG Wallace RB Shadyab AH Kroenke CH . Association of major dietary protein sources with all-cause and cause-specific mortality: prospective cohort study. J Am Heart Assoc. (2021) 10:e015553. doi: 10.1161/JAHA.119.015553, 33624505 PMC8174240

[43] Um CY Judd SE Flanders WD Fedirko V Bostick RM. Associations of calcium and dairy products with all-cause and cause-specific mortality in the REasons for geographic and racial differences in stroke (REGARDS) prospective cohort study. Nutr Cancer. (2017) 69:1185–95. doi: 10.1080/01635581.2017.1367946, 29125314 PMC6145131

[44] Cruijsen E Jacobo Cejudo MG Küpers LK Busstra MC Geleijnse JM. Dairy consumption and mortality after myocardial infarction: a prospective analysis in the alpha omega cohort. Am J Clin Nutr. (2021) 114:59–69. doi: 10.1093/ajcn/nqab026, 33826695 PMC8246616

[45] Miyagawa N Takashima N Harada A Kadota A Kondo K Miura K . Dairy intake and all-cause, cancer, and cardiovascular disease mortality risk in a large Japanese population: a 12-year follow-up of the J-MICC study. J Atheroscler Thromb. (2025) 32:596–607. doi: 10.5551/jat.65049, 39537182 PMC12055505

[46] Stefler D Landstra E Bobak M. Household availability of dietary fats and cardiovascular disease and mortality: prospective evidence from Russia. Eur J Pub Health. (2021) 31:1037–41. doi: 10.1093/eurpub/ckab128, 34329405 PMC8565488

[47] Huang LY Wahlqvist ML Huang YC Lee MS. Optimal dairy intake is predicated on total, cardiovascular, and stroke mortalities in a Taiwanese cohort. J Am Coll Nutr. (2014) 33:426–36. doi: 10.1080/07315724.2013.875328, 25078873

[48] Praagman J Dalmeijer GW Van Der Schouw YT Soedamah-Muthu SS Monique Verschuren WM Bas Bueno-de-Mesquita H . The relationship between fermented food intake and mortality risk in the European prospective investigation into cancer and nutrition-Netherlands cohort. Br J Nutr. (2015) 113:498–506. doi: 10.1017/S0007114514003766, 25599866

[49] Lin P Gui X Liang Z Wang T. Association of yogurt and dietary supplements containing probiotic consumption with all-cause and cause-specific mortality in US adults: a population-based cohort study. Front Nutr. (2022) 9:803076. doi: 10.3389/fnut.2022.803076, 35198588 PMC8858963

[50] Tutunchi H Naghshi S Naemi M Naeini F Esmaillzadeh A. Yogurt consumption and risk of mortality from all causes, CVD and cancer: a comprehensive systematic review and dose-response meta-analysis of cohort studies. Public Health Nutr. (2023) 26:1196–209. doi: 10.1017/S1368980022002385, 36349966 PMC10346031

[51] Rizzoli R Biver E. Role of fermented dairy products in the health benefits of a mediterranean diet. Aging Clin Exp Res. (2024) 36:75. doi: 10.1007/s40520-024-02721-x, 38502263 PMC10950975

[52] Yilmaz B Alvanoudi P Kalogeropoulou A Santa D Bulmuş-Tüccar T Nikolaou A . Fermented dairy product consumption and blood lipid levels in healthy adults: a systematic review. Front Nutr. (2025) 12:12. doi: 10.3389/fnut.2025.1651134, 41019545 PMC12461264

[53] Companys J Pla-Pagà L Calderón-Pérez L Llauradó E Solà R Pedret A . Fermented dairy products, probiotic supplementation, and cardiometabolic diseases: a systematic review and meta-analysis. Adv Nutr. (2020) 11:834–63. doi: 10.1093/advances/nmaa030, 32277831 PMC7360468

[54] Anto L Warykas SW Torres-Gonzalez M Blesso CN. Milk polar lipids: underappreciated lipids with emerging health benefits. Nutrients. (2020) 12:1001. doi: 10.3390/nu12041001, 32260440 PMC7230917

[55] Lordan R Vidal NP Huong Pham T Tsoupras A Thomas RH Zabetakis I. Yoghurt fermentation alters the composition and antiplatelet properties of milk polar lipids. Food Chem. (2020) 332:127384. doi: 10.1016/j.foodchem.2020.127384, 32615384

[56] Vors C Joumard-Cubizolles L Lecomte M Combe E Ouchchane L Drai J . Milk polar lipids reduce lipid cardiovascular risk factors in overweight postmenopausal women: towards a gut sphingomyelin-cholesterol interplay. Gut. (2020) 69:487–501. doi: 10.1136/gutjnl-2018-318155, 31189655 PMC7034342

[57] Venkat M Chia LW Lambers TT. Milk polar lipids composition and functionality: a systematic review. Crit Rev Food Sci Nutr. (2024) 64:31–75. doi: 10.1080/10408398.2022.2104211, 35997253

[58] Chen J Zhang Y Liu Z Wang J Zhang S Shao J . Comprehensive characterization and comparison of polar lipids in bovine and donkey milk based on lipidomics. Food Chem. (2025) 496:146801. doi: 10.1016/j.foodchem.2025.146801, 41166817

[59] Zhang M Dong X Huang Z Li X Zhao Y Wang Y . Cheese consumption and multiple health outcomes: an umbrella review and updated meta-analysis of prospective studies. Adv Nutr. (2023) 14:1170–86. doi: 10.1016/j.advnut.2023.06.007 37328108 PMC10509445

[60] Uppsala Universitet Milk puts women at risk of cardiovascular disease. Uppsala University. 2024. Available online at: https://www.uu.se/en/news/2024/2024-11-27-milk-puts-women-at-risk-of-cardiovascular-disease (Accessed October 6, 2025).

