# Supplementary material for: The dose-response relationship between dairy product intake and all-cause and cardiovascular mortality risk: a systematic review and meta-analysis of prospective cohort studies
Source: Front Nutr. 2026 Jan 22;13:1731841. doi: 10.3389/fnut.2026.1731841 (PMC12873575; doi:10.3389/fnut.2026.1731841)
Supplement: Supplementary file 1 [file Table_1.docx]

Supplementary Material

**The dose-response relationship between dairy product intake and all-cause and cardiovascular mortality risk: A systematic review and meta-analysis of prospective cohort studies**

**[1 Supplementary Table 1 Literature Search Strategy for PubMed, EMBASE and Web of Science Databases 1](#_Toc32264)**

**[2 Supplementary Figure 1 A-D Risk of publication bias analysis 2](#_Toc11365)**

**[3 Supplementary Table 2 Risk of bias judgements for each domain (from the ROBINS-E detailed guidance) 3](#_Toc20341)**

**[4 Supplementary Table 3 Study characteristics 5](#_Toc25019)**

**[5 Supplementary Table 4 Grade Assessment for Dose-Response Analysis of Dairy Product Exposures and All-Cause or Cardiovascular Mortality 15](#_Toc26296)**

**[6 Supplementary Figure 2 A-H Sensitivity Analysis Plot  16](#_Toc22982)**

**[7 Supplementary Figure 3 Sensitivity Analysis Plot (Total Dairy Intake vs. CVD Mortality: Including the Study by Lin-Yuan Huang et al., 2014) 17](#_Toc6718)**

**[9 Supplementary Table 5 Results of meta-regression 18](#_Toc27869)**

**[Supplementary Table 5.1 Results of univariate meta-regression analyses for milk-CVD mortality association 18](#_Toc22046)**

**[Supplementary Table 5.2 Results of multivariate meta-regression analysis for milk-CVD mortality association (including Sex, Region, Median Follow up Years, Total number) 18](#_Toc24430)**

**[Supplementary Table 5.3 Results of univariate meta-regression analyses for total dairy-CVD mortality association 19](#_Toc31478)**

**[Supplementary Table 5.4 Results of multivariate meta-regression analysis for total dairy-CVD mortality association(including Sex, Region, Median Follow up Years, Total number) 19](#_Toc11744)**

**[9 Supplementary Figure 4 Forest Plot of Subgroup Analyses for Milk and Total Dairy Intake in Association with Cardiovascular Disease (CVD) Mortality 20](#_Toc16592)**

# **1 Supplementary Table 1** Literature Search Strategy for PubMed, EMBASE and Web of Science Databases

| **PubMed** | **EMBASE** | **WEB OF SCIENCE** |
| --- | --- | --- |
| **( ( ("dairy products"[MeSH] OR dairy[tiab] OR milk[tiab] OR "milk consumption"[tiab]) OR (cheese[tiab] OR yogurt[tiab] OR butter[tiab] OR kefir[tiab] OR cream[tiab]) OR ("fermented milk"[tiab] OR "dairy fat"[tiab] OR whey[tiab] OR casein[tiab] OR "dairy intake"[tiab]) ) AND ( mortality[tiab] OR death[tiab] OR survival[tiab] OR "all cause mortality"[tiab] OR "cardiovascular death"[tiab] OR "CVD mortality"[tiab] OR "cardiac mortality"[tiab] OR "heart disease mortality"[tiab] OR "myocardial infarction death"[tiab] ) AND ( cohort[tiab] OR prospective[tiab] OR "follow up"[tiab] OR "observational study"[tiab] OR "population-based"[tiab] OR "risk assessment"[tiab] ) )** | **('dairy product'/exp OR 'dairy product' OR 'milk'/exp OR 'milk' OR 'cheese'/exp OR 'cheese' OR 'yogurt'/exp OR 'yogurt' OR dairy:ti,ab,kw OR milk:ti,ab,kw OR cheese:ti,ab,kw OR yogurt:ti,ab,kw OR kefir:ti,ab,kw) AND ('mortality'/exp OR 'mortality' OR 'cause of death'/exp OR 'cause of death' OR 'all cause mortality':ti,ab,kw OR 'cardiovascular death':ti,ab,kw OR 'cardiac death':ti,ab,kw) AND ('cohort analysis'/exp OR 'cohort analysis' OR 'prospective study'/exp OR 'prospective study' OR cohort*:ti,ab,kw OR prospective:ti,ab,kw OR 'follow up':ti,ab,kw) AND [humans]/lim** | **TS=(( (dairy OR milk OR cheese OR yogurt) OR (kefir OR "milk products" OR "dairy fat") OR (curd OR "cultured milk")) AND ("mortality" OR "death" OR "survival" OR "cardiovascular death" OR "cardiac death" OR "CVD mortality" OR "heart disease mortality" OR "myocardial infarction death") AND ("cohort" OR prospective OR "follow up" OR "observational study" OR "population-based"))** |
| June 19, 2025: 1432 | June 19, 2025: 1647 | June 19, 2025: 1718 |

**2 Supplementary Figure 1 A-D** Risk of publication bias analysis


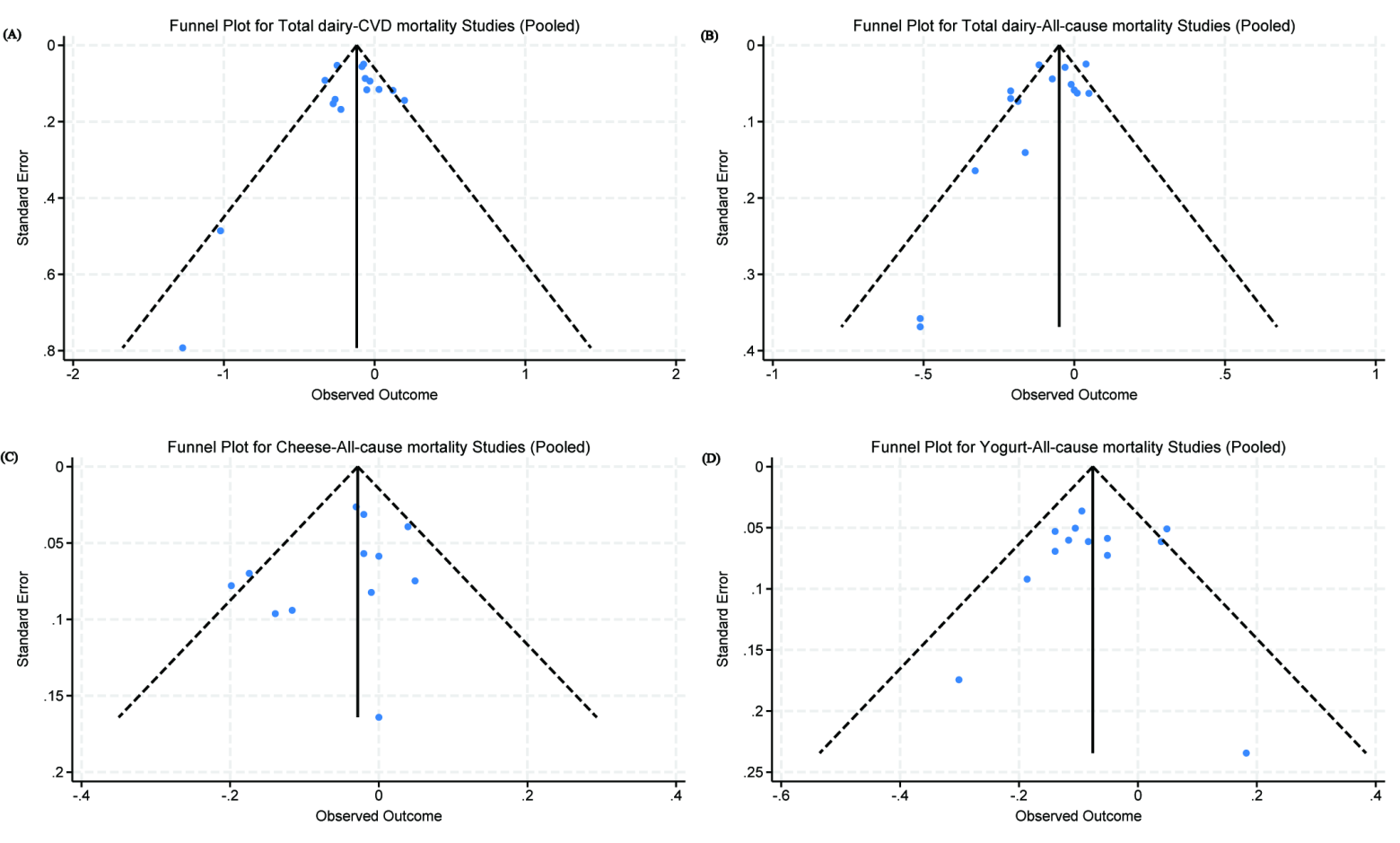


**3 Supplementary Table 2** Risk of bias judgements for each domain (from the ROBINS-E detailed guidance)

| **Study** | **Bias due to confounding** | **Bias due to exposure assessment** | **Bias due to selection of participants** | **Bias due to misclassification during follow-up** | **Bias due to missing data** | **Bias due to measurement of the outcome** | **Bias due to selective reporting of the results** | **Overall judgement** |
| --- | --- | --- | --- | --- | --- | --- | --- | --- |
| Xin-Yan Wang(2020) | Moderate | Moderate | Low | Low | Low | Low | Low | **Moderate** |
| Mahshid Dehghan（2018） | Moderate | Moderate | Low | Low | Low | Low | Low | **Moderate** |
| Sanyu Ge（2023） | Moderate | Moderate | Low | Low | Low | Low | Low | **Moderate** |
| Valeria Pala（2019） | Moderate | Low to moderate | Low | Low | Low | Low | Low | **Moderate** |
| Yu Zhang（2025） | Moderate | Low to moderate | Low | Low | Low | Low | Low | **Moderate** |
| Sabita S. Soedamah-Muthu（2013） | Moderate | Moderate | Low | Low | Low | Low | Low | **Moderate** |
| Jimmy Chun Yu Louie（2013） | Moderate | Moderate | Low | Low | Low | Low | Low | **Moderate** |
| Fernanda Marcelina Silva（2022） | Moderate | Low to  moderate | Low | Low | Low | Low | Low | **Moderate** |
| Shanjie Wang（2021） | Moderate | Moderate | Low | Low | Low | Low | Low | **Moderate** |
| Wang, Xiang Jun（2020） | Moderate | Moderate | Low | Low | Low | Low | Low | **Moderate** |
| Zhengjun Lin（2025） | Moderate | Moderate | Low | Low | Low | Low | Low | **Moderate** |
| Mohammad Talaei（2017） | Moderate | Moderate | Low | Low | Low | Low | Low | **Moderate** |
| Daniela Schmid（2020） | Moderate | Moderate | Low | Low | Low | Low | Low | **Moderate** |
| Jing Guo (2022) | Moderate | Moderate | Low | Low | Low | Low | Low | **Moderate** |
| Xiaona Na(2022) | Moderate | Low to moderate | Low to moderate | Low | Low to moderate | Low | Low | **Moderate** |
| Yukai Lu (2022) | Moderate | Moderate | Low | Low | Low | Low | Low | **Moderate** |
| Yangbo Sun (2021) | Moderate | Moderate | Low | Low | Low | Low | Low | **Moderate** |
| Caroline Y. Um（2017） | Moderate | Moderate | Low | Low | Low | Low | Low | **Moderate** |
| Goldbohm R Alexandra（2011） | Moderate | Low | Low | Low | Low | Low | Low | **Moderate** |
| Esther Cruijsen(2021) | Moderate | Moderate | Low | Low | Low | Low | Low | **Moderate** |
| M Bonthuis (2010) | Moderate | Low | Moderate | Low | Low | Low | Low | **Moderate** |
| Emily Sonestedt (2021) | Moderate | Low | Low | Low | Low | Low | Low | **Moderate** |
| Maryam S. Farvid（2017） | Moderate | Moderate | Low | Low | Low | Low | Low | **Moderate** |
| Naoko Miyagawa（2025） | Moderate | Low | Low | Low | Low | Low | Low | **Moderate** |
| Denes Stefler（2021） | Moderate | Moderate | Moderate | Low | Low | Moderate | Low | **moderate** |
| Karl Michaëlsson professor（2014） | Moderate | Low | Moderate | Low | Low | Low | Low | **Moderate** |
| Karl Michaëlsson (2020) | Moderate | Low | Low | Low | Low | Low | Low | **Moderate** |
| Lin-Yuan Huang（2014） | Moderate | Low | Low | Low | Low | Low | Low | **Moderate** |
| Jaike Praagman (2015) | Moderate | Moderate | Low | Moderate | Low | Low | Low | **Moderate** |

**4 Supplementary Table 3 Study characteristics**

| **First author**  **(year)** | **Country** | **Date source** | **Mean/median age and age range** | **% Men** | **Total number** | **Median Follow up Years** | **Exposure** | **Exposure measure** | **Conversion Factors** | **HR RR OR** | **Outcome assessment** | **Adjustment** |
| --- | --- | --- | --- | --- | --- | --- | --- | --- | --- | --- | --- | --- |
| Xin-Yan Wang (2020) | China | China-PAR project  (China MUCA 1998,  InterASIA, CIMIC) | 18-74y | 39.17 | 91,757 | 5.8y | Milk | Simplified FFQ | All intake values and specific doses (e.g., 0, 43.78, 229.73 g/day) are reported in grams and used directly. | HR | Interviews, hospital records, death certificates | Age, sex, geographic region, residential area, education level，family history of CVD, smoking, alcohol drinking, physical activity level, BMI, healthy diet status，systolic blood pressure, fasting blood glucose, total cholesterol, HDL-C. |
| Mahshid Dehghan （2018） | 21 countries across five continents | Prospective Urban  Rural Epidemiology  (PURE) study | 35–70y | 41.7 | 136,384 | 9.1y | Total dairy Milk Yoghurt Cheese Butter | Country-specific validated FFQ | 1 serving of milk = 244 g; 1 serving of yoghurt = 244 g; 1 serving of cheese = 15 g; 1 serving of butter = 5 g (as defined in the study questionnaire) | HR | Standard case-report forms and adjudicated centrally by trained physicians | Age, sex, education, urban or rural location, smoking status, physical activity, history of diabetes, family history of cardiovascular disease, family history of cancer, quintiles of fruit and vegetable intake, red meat intake, starchy foods intake, and total energy intake. Centre included as a random effect. Additional adjustments for specific analyses (e.g., saturated fatty acid models adjusted for protein intake and vice versa). |
| Sanyu Ge （2023） | Japan | Japan Public Health Center-based prospective  (JPHC) study | 40-69y | 46.2 | 93310 | 19.3y | Total dairy Milk Cheese | 147-item FFQ | Sex-specific intake quartiles directly reported in grams for each dairy category. Males: Total dairy (7.6, 59.5, 153.9, 321.3 g/day); Milk (0.3, 29.1, 117.2, 272.8 g/day); Cheese (0, 1, 1.6, 6.1 g/day).  Females:​ Total dairy (25.5, 105.1, 198.8, 375.4 g/day); Milk (1.2, 57.5, 142.9, 305.4 g/day); Cheese (0, 1, 1.8, 7.6 g/day). | HR | Death certificates and residential registry | Age, study area，smoking, alcohol, BMI, physical activity, hypertension, diabetes, green tea, coffee, vegetable/fruit intake, total energy/fat, female-specific factors (menopausal status, exogenous hormones)，other dairy intake (for specific dairy analyses). |
| Valeria Pala （2019） | Italy | European Prospective  Investigation  into Cancer  and Nutrition  (EPIC)–Italy cohort | 50.1y | M+F | 45,009 | 14.9y | Milk Yogurt Cheese Butter | Validated semiquantitative FFQs | Dose categories were directly reported as ranges in grams: milk(0, >0-50, 50-160, 160-200, >200 g/day). yogurt( 0, >0-35, 35-120, >120 g/day)、cheese(0-28, 28-70, 70-100, >100 g/day)、butter（0, >0-1.4, >1.4-100.1 g/day） | HR | Linkage to mortality databases | Stratified by center, age, and sex, adjusted for energy intake (excluding alcohol), weight, height, waist-to-hip ratio, alcohol consumption, smoking status, physical activity, socioeconomic status (RII), dietary fiber, Italian Mediterranean Index, and sugar intake (for milk and yogurt models). |
| Yu Zhang （2025） | USA | Nurses’  Health Study  (1990-2023),  Nurses’  Health Study II  (1991-2023), and Health  Professionals Follow-up Study  (1990-2023) | 30-75y | 13.3 | 221,054 | 33y | Butter | Validated semiquantitative FFQ | Median intake values directly reported in g/day for each sex: Males (0.1, 4.1, 8.1, 14.0g/day); Females (0.1, 4.8, 8.8, 13.1g/day). No conversion needed. | HR | National Death Index and medical records | Age, calendar time, total energy intake, menopausal status and hormone use (women), race, BMI, alcohol consumption, smoking status, physical activity, Alternative Healthy Eating Index, aspirin and multivitamin use, baseline histories of hypertension and hypercholesterolemia, family history of myocardial infarction, cancer, and diabetes; mutual adjustment for butter and plant-based oil intake. |
| Sabita S. Soedamah-Muthu （2013） | UK | Whitehall II study | 35–55y | 72 | 4526 | 11.7y | Total dairy Yogurt Cheese | 114-item semiquantitative FFQ | Median intakes directly reported in grams:  Total dairy:246, 371, 575 g/day  Yogurt: 0, 21, 117 g/day  Cheese:6, 17, 31 g/day | HR | National Health Service registry | Age, ethnicity, employment grade,smoking, alcohol, BMI, physical activity, family history of CHD/hypertension  ,fruit, vegetables, bread, meat, fish, coffee, tea, total energy intake. |
| Jimmy Chun Yu Louie （2013） | Australia | Blue Mountains  Eye Study (BMES) | 49–97y | 43.4 | 2662 | 15y | Total dairy | 145-item validated semi-quantitative FFQ | Intake originally reported in serves/day (Australian Dietary Guidelines standard: 1 serve dairy = 250 g). Median intakes were: T1=0.6 serves , T2=1.4 serves , T3=2.9 serves . | HR | Linkage to Australian National Death Index | Age, sex, total energy intake, baseline BMI, weight change during follow-up, physical activity, previous acute myocardial infarction, previous stroke, smoking status, stage II hypertension, type 2 diabetes status, use of antihypertensive medication and statins, and change in dairy intake. |
| Fernanda Marcelina Silva （2022） | Brazil | Brazilian  Longitudinal  Study of  Adult Health  (ELSA-Brasil) | 35–74y | 49.9 | 6671 | 8.1y | Total dairy Milk | 114-item semi-quantitative FFQ | For both total dairy and milk, the representative dose for each quartile was calculated as the average of the midpoints of the sex-specific intake ranges.  Total dairy:145.1, 267.0, 420.3, 630.45 g/day.  Milk:45, 179.6, 290.4, 320.4 g/day. | HR | Annual follow-up calls, hospital records, and linkage to the Ministry of Health mortality system | Sociodemographic characteristics (sex, age, educational level), behaviors and health indicators (physical activity, smoking, excessive alcohol use, fruit and vegetable intake, BMI), and comorbidities (diabetes mellitus, hypertension, hypercholesterolemia). |
| Shanjie Wang （2021） | USA | National Health and Nutrition Examination  Survey  (NHANES)  1999–2014 | 46.7y | 48.7 | 29,283 | 8.3y | Whole milk Low-fat milk | Standardized questionnaires (30-day recall of milk type and frequency) and 24-hour dietary recalls | Milk consumption was directly reported in grams per day (g/d) for each fat-content category. The reported mean±SE values are: Whole-fat (354.8±7.05), 2g/100g Low-fat (320.5±4.53), ≤1g/100g Low-fat (340.8±5.74), Other types (235.7±17.12) g/d. | HR | Linkage to National Death Index | Age, sex, race/ethnicity,education, marital status, income, BMI, lifestyle factors,comorbidities, lipid levels, eGFR,total energy, macronutrients, fruit/vegetable and red meat intake. |
| Wang, Xiang Jun （2020） | China | Guangzhou Biobank Cohort Study (GBCS) | 62.8y | 28.5 | 18214 | 11.5y | Milk | 282-item FFQ | Milk intake was categorized as: 0 (non-consumer), 1-3, and3+portions/week, with 1 portion = 250 ml. For dose-response analysis, the following representative daily intakes (g/day) were assigned to each group: 0, 89.3, 125g/d. | HR | Linkage to Guangzhou Center for Disease Control and Prevention records | Sex, age, family income, education, occupation, smoking status, alcohol use, physical activity, BMI, self-rated health, diabetes, hypertension, hyperlipidemia, daily dietary energy intake, and dietary patterns (aMED, DASH, aHEI-2010). |
| Zhengjun Lin（2025） | UK | UK Biobank | 37-73y | 44.5 | 186168 | 13.4y | Full-fat yoghurt low-fat yoghurt | Oxford WebQ (24-hour dietary recall questionnaire) | Both full-fat and low-fat yogurt were analyzed using identical daily intake categories: <50, 50-100, and >100 g/d | HR | Death certificates from national health services | Sociodemographic factors (sex, age, ethnicity, education, Townsend deprivation index), lifestyle factors (smoking, alcohol consumption, physical activity, BMI), health indicators (hypertension, diabetes, high cholesterol, long-standing illness, drug use), and dietary factors (total energy, total fat, fruit, vegetables, red/processed meat, other dairy, sugar-sweetened beverages, etc.). |
| Mohammad Talaei （2017） | Singapore | Singapore Chinese  Health  Study  (SCHS) | 45–74y | 41.8 | 60,298 | 14.8y | Total dairy | 165-item validated semiquantitative FFQ | Median total dairy intake (with interquartile range, IQR) was directly reported in g/ day for each group. No conversion was applied. The reported values were: 1.32 (0.51–2.39), 14.1 (8.08–16.0), 37.6 (28.9–47.2), 252 (127–270) g/day. | HR | Linkage to Singapore's nationwide death registry | Age, sex, dialect, interview year, education, BMI, physical activity, smoking, alcohol use, baseline history of diabetes/hypertension/CHD/stroke, total energy intake, and dietary intakes of red meat, poultry, fish, vegetables, fruits, grains, tea, and coffee. |
| Daniela  Schmid （2020） | USA | Nurses’ Health Study (NHS) and Health Professionals Follow-Up Study (HPFS) | 30-75y | 32.8 | 122,626 | 30y | Yogurt | Updated validated semiquantitative FFQs | Yogurt consumption frequency was categorized as: Never, >0 to ≤1–3/month, 1/week, 2–4/week, and >4/week, with 1 serving defined as 245 g. For dose-response analysis, the following representative daily intakes (g/day) were assigned: 0, 17.5, 35, 87.5, 175. | HR | State statistics, National Death Index, and medical records | Age, follow-up cycle, height, BMI, BMI at 18/21 years, ethnicity, physical activity, smoking, pack-years, history of hypertension/hypercholesterolemia/diabetes, family history of diseases, multivitamin/aspirin use, menopausal status and hormone use (women), total energy, alcohol, glycemic load, and intakes of red meat, processed meat, nuts, fruits, vegetables, fiber, and calcium. |
| Jing Guo (2022) | Denmark | WHO-initiated survey Monitoring of Trends and Determinants in Cardiovascular Diseases (MONICA) cohort | 30–60 y | 47.8 | 1746 | 30y | Total dairy | 7-day weighed diet diary | Total dairy intake was analyzed in quartiles (Q1-Q4) of weekly consumption (g/wk). The mean (SD) values for each quartile, as reported in Table 2, are: Q1: 119.6 (48.1), Q2: 253.3 (36.4), Q3: 391.1 (46.3), Q4: 697.0 (223.1) g/wk. | HR | Linkage to the National Patient Registry with notification of death certificate received automatically | Gender, BMI, food energy intake, alcohol consumption, education, smoking, physical activity, family history of MI, multivitamin. Model 1 plus serum total cholesterol, serum triacylglycerols, incidence of hypertension. |
| Xiaona Na (2022) | China | China Health and Nutrition Survey (CHNS) data from 1997-2015 | **Not specified** | M+F | 14738 | 10.31y | Milk | Three day 24-h dietary recall. | Milk intake was categorized into three groups: No Consumption, 0.1–2 Portions/Week, and >2 Portions/Week. One portion was defined as 300 grams. | HR | Information reported in each wave of the China Health and Nutrition Survey (CHNS) | Age, sex, education, place of residence, individual annual income ,,further adjusted for smoking status, alcohol intake, physical activity, BMI, chronic disease history, vegetable intake, fruit intake, red meat intake, dietary diversity score (DDS), energy intake,inverse probability of treatment weighting, which weights participants based on the probability of being in a specific milk intake group, balancing confounding factors across groups. |
| Yukai Lu (2022) | Japan | Miyagi Cohort Study initiated in 1990 | 40–64 y | 48.5 | 34161 | 25y | Total dairy  Milk  yogurt  Cheese | Validated FFQ | Sex-specific mean (SD) dairy intakes (g/day) as reported:  Men: Q1: 6.6 (7.5), Q2: 71.5 (27.7), Q3: 180.0 (41.8), Q4: 229.7 (26.7)  Women:​ Q1: 17.5 (19.0), Q2: 103.8 (32.1), Q3: 212.9 (2.6), Q4: 250.1 (27.0)  These intake data were assessed using a validated food frequency questionnaire (FFQ), with Spearman correlation coefficients against 3-day diet records ranging from 0.36 (cheese) to 0.72 (milk) in men, and 0.36 (cheese) to 0.65 (milk) in women. | HR | Death certificates | Age,age plus education level, BMI, smoking status, alcohol drinking status, history of hypertension and diabetes,energy intake, vegetable and fruit intake, fish intake. |
| Yangbo Sun (2021) | USA | Enrolled from 1993-1998, followed up until February 2017 | 50-79y | 0 | 102521 | 18.1y | Total dairy | The WHI-FFQ | The reported values for total dairy intake, possibly in cup-equivalents per day, are: 0.5, 0.9, 1.4, 2.0, and 3.3.Conversion to grams was based on the USDA standard: 1 cup = 240 g. | HR | Reviewing death certificates, medical records, autopsy reports, or linkage to the National Death Index | Age, race/ethnicity, education, income, Observational Study/Clinical Trials, hormone use history, smoking status, physical activity, alcohol intake, total energy intake, baseline health status, family history of heart attack/stroke, whole grain/vegetable/fruit/sugar-sweetened beverage consumption, mutual adjustment for other protein sources ,BMI. |
| Caroline Y. Um （2017） | USA | Recruitment: January 2003- October 2007; Follow-up until December 31, 2012 | ≥45 y | 44.1 | 21427 | 7.6y | Total dairy  Whole milk | Block 98 FFQ | Sex- and quintile-specific median (range) intakes of dairy and whole milk (g/d):  Total dairy  Men (Quintiles 1–5): 22.2 (0–<46.8), 79.8 (46.8–<116), 154 (116–<206), 268 (206–<350), 511 (350–1,468)  Women (Quintiles 1–5): 17.0 (0–<37.1), 31.4 (37.1–<93.8), 133 (93.8–<177), 242 (177–<319), 484 (319–1,368)  Whole milk  Men (Quintiles 1–5): 0 (0), 10.9 (>0–<34.8), 74.2 (34.8–<112), 171 (112–<246), 382 (246–1,216)  Women (Quintiles 1–5): 0 (0), 10.4 (>0–<20.9), 41.9 (20.9–<78.0), 119 (78.0–<186), 325 (186–1,288) | HR | Death certificates and National Death Index | Age, sex, race, region, body mass index, smoking status, alcohol intake, physical activity, nonsteroidal anti-inflammatory drug (NSAID) and aspirin use, hormone replacement therapy use (females), education, annual income, supplemental calcium, total energy intake, fruit and vegetable intake, processed and red meat intake, dietary oxidative balance score. |
| Goldbohm R  Alexandra （2011） | The Netherlands | Netherlands Cohort Study (NLCS) 1986-1996 | 55–69y | 48.2 | 120852 | 10y | Cheese | FFQ | Median cheese intake values were directly reported as 1, 11, 19, 37, and 56 g/d. | RR | Dutch Central Bureau of Genealogy and Statistics | Age, education, smoking (status, number, duration), BMI, nonoccupational/occupational physical activity, multivitamin use, alcohol, energy, energy-adjusted mono- and polyunsaturated fats, vegetable and fruit consumption. |
| Esther  Cruijsen (2021) | The Netherlands | Alpha Omega Cohort (2002–2018) | 60–80y | 79 | 4365 | 12 y | Yogurt Cheese Total dairy | FFQ | Reported median intake (g/d) for combined-sex groups, used directly:  Total Dairy (Q1-Q4):135.8, 245.6, 343.8, 527.5  Cheese (T1-T3):12.6, 36.2, 61.3  Yogurt: 8.1, 36.0, 87.5 | HR | Municipal registries, national mortality registry, treating physicians, and family members | Total energy intake, age, sex,physical activity, smoking, alcohol intake, diabetes, obesity,intakes of whole grains, refined grains, fruits, vegetables, red meat, processed meat, sugar-sweetened beverages, coffee, tea, fish, sodium from foods; cheese analysis additionally adjusted for sodium from foods other than cheese,DHD15-index. |
| M Bonthuis (2010) | Australia | Nambour Skin Cancer Prevention Trial Cohort | 25–78y | M+F | 1529 | 14.4y | Total dairy Milk Yoghurt | FFQ | The following representative daily intake levels, all reported in grams and used directly in the dose-response analysis, were:  Total dairy:​ 174, 332, 599 g/day  Milk:109, 250, 500 g/day  Yogurt: 0, 11, 76 g/day | HR | National Death Index | Age (as time scale), sex, total energy intake,BMI, alcohol intake, school leaving age, physical activity level, pack years of smoking, dietary supplement use, presence of any medical condition, β-carotene treatment,calcium,dietary calcium intake medications for hypertension, diabetes, cardiac disorder, and β-adrenergic blocking agents. |
| Emily Sonestedt (2021) | Sweden | Malmö Diet and Cancer (MDC) cohort | 45-73y | 38 | 26190 | 19y | Cheese Butter | FFQ | Cheese intake was categorized into six groups (g/day): 0–20, 20–40, 40–60, 60–80, 80–100, >100.  Cheese intake was categorized into six groups (g/day): 0, 0-10, 10–20, 20–30, 30–40, 40–50, >50. | HR | The National Tax Board | Age, sex, season, diet assessment method, energy intake (continuous), BMI (continuous), smoking habits, alcohol habits, educational status, leisure-time physical activity, intake of fruit and vegetables (continuous), meat (continuous), fiber (continuous), and sugar-sweetened beverages (continuous). |
| Maryam S. Farvid （2017） | Iran | Golestan Cohort Study（2004 - 2015） | 36–83y | 43 | 42403 | 8y | Total dairy Milk Yogurt Cheese | 116-item FFQ | Serving sizes were defined as: milk/yogurt = 230 g, cheese = 28 g.  Reported median intake (servings/day) across intake groups:  Total dairy：0.4、0.8、1.2、1.6、2.4  Milk:0, 0.04, 0.1, 0.3, 0.6  Yogurt: 0.1, 0.3, 0.4, 0.6, 0.9  Cheese:0, 0.1, 0.3, 0.5, 0.8 | HR | Annual follow-up, family reports, physician visits, verbal autopsy questionnaires | Age, sex, ethnicity, education, marital status, residence, smoking, opium use, alcohol use, BMI, systolic blood pressure, occupational physical activity, family history of cancer, wealth score, medication use, and total energy intake,fruits, vegetables, grains, and total red meat. |
| Naoko Miyagawa （2025） | Japan | Japan Multi-Institutional Collaborative Cohort (J-MICC) study | 35–69y | 42.8 | 79715 | 12.4y | Total dairy Milk Yogurt | FFQ | The mean daily intake (with range) for each dairy product was directly reported in grams per day (g/d):  Total dairy:male：10(0,16)、66(42,96)、170(160,260);Female:26(10,50)、128(98,155)、235(178,255)  Milk:male：0(0,0)、32(16,32)、160(128,160);Female:0(0,15.5)、78(31,78)、155(155,155)  Yogurt:male：0(0,0)、10(10,10)、80(50,180);Female:10(0,10)、20(20,50)、100(80,100) | HR | Based on vital status from local government records and National Vital Statistics | Age and study site,history of hypertension, diabetes, dyslipidemia, and BMI categories,smoking status, drinking status, leisure-time physical activity, and intake of red meat, fish, vegetables, and fruits. |
| Denes Stefler （2021） | Russia | Russian Longitudinal Monitoring Survey (RLMS) | ≥18y | 39.2–45.2 | 6618 | 11y | Butter | Household Budget Surveys (HBSs) | Based on household butter purchase (median = 0.33 kg/person/week), three dose groups were created. Their representative daily intakes (g/day) were assigned as: 0 (No purchase), 23.6 (Low purchase: midpoint of 0-0.33), and 94.3 (High purchase: 2 × median). | OR | Self-reported (reported by other household members) | Age and sex,education, alcohol intake frequency, smoking habits,the availability of the other three dietary fats and oils. |
| Karl Michaëlsson professor （2014） | Sweden | Swedish Mammography Cohort and Cohort of Swedish Men | 39-79y | 42.7 | 106772 | 22y | Milk | FFQ | Milk intake was analyzed in four categories:<1 glass (<200 g/d) 1-2 glasses (200-399 g/d) 2-3 glasses (400-599 g/d) ≥3 glasses (≥600 g/d).Additionally, a continuous dose-response analysis was performed per 200 g/day increment, which corresponds to one glass of milk.  The analysis compared two extreme intake levels: less than one glass per day (mean: 60 g/day) and three or more glasses per day (mean: 680 g/day). | HR | Swedish cause of death registry | Age, body mass index, height, total energy intake, alcohol intake, healthy dietary pattern, calcium and vitamin D supplementation, ever use of cortisone, educational level, living alone, physical activity, smoking status, Charlson’s comorbidity index; women additionally adjusted for oestrogen replacement therapy and nulliparity,nutrients (calcium, vitamin D, etc.) and other dairy products. |
| Michaëlsson and Byberg (2020) | Sweden | Swedish Mammography Cohort (SMC) | 53.2–54.1y | 0 | 61433 | 23y | Non-fermented milk Total non-fermented milk | FFQ | Non-fermented Milk/Total non-fermented milk intake categories were originally reported in mL/day: <200, 200–399, 400–599, ≥600. For analysis, a standard density of 1 g/mL was assumed for conversion to grams where applicable. | HR | Swedish Total Population Register | Age, body mass index, height, total energy intake, total alcohol intake, healthy dietary pattern, calcium and vitamin D supplementation, ever use of cortisone, educational level, living alone, physical activity level, smoking status, estrogen replacement therapy, nulliparity, Charlson’s comorbidity index,the intakes of the other milk fat subtypes (for specific fat types); for total milk, adjusted for total intake of saturated fat. |
| Lin-Yuan Huang （2014） | Taiwan | Nutrition and Health Survey in Taiwan (NAHSIT,1993-1996) linked to national death registration (1993–2008) | 19–64y | 48.3 | 3810 | 13.7y | Total dairy | FFQ | Dairy intake was categorized by consumption frequency (times/week) as shown: 0, 0.1-3.0, 3.1-7.0, >7.0. Mean ± SE and range of total dairy 0、1.38 ± 0.04 、5.01 ± 0.12 、10.3 ± 0.33For dose-response analysis, "1 time" was equated to "1 standard serving" based on the Taiwanese dietary context (milk as the primary dairy). One serving was defined as 240 g. The reported weekly frequencies were thus converted to estimated daily gram intake for analysis. | HR | International Classification of Diseases | Age, gender, BMI, region, ethnicity, education level, marriage, and history of disease,smoking, drinking, betel nut chewing, and supplement use,Overall Dietary Index–Revised (dairy score excluded),calcium intake,vitamin D intake, consumption of low-fat or skim milk. |
| Jaike Praagman (2015) | The Netherlands | Singapore Chinese Health Study (SCHS) | 20–70y | 26 | 34409 | 15y | Cheese Yogurt | A validated FFQ | The median daily intake for yogurt and cheese was directly reported in the original study as follows (g/d):Yogurt: 3.8 , 26.6 , 62.9 , 144.5  Cheese:6.6,19.6,31.8,53.2 | HR | National mortality register | Age, sex, total energy intake,physical activity, education level, hypertension, smoking, BMI,fruit, vegetables, alcohol intake; fermented vegetables adjusted for total vegetables excluding fermented ones. |

**5 Supplementary Table 4** Grade Assessment for Dose-Response Analysis of Dairy Product Exposures and All-Cause or Cardiovascular Mortality

| **No. of studies** | **Design** | **Risk of bias** | **Inconsistency** | **Indirectness** | **Imprecision** | **Other** | **Cases** | **Sample size** | **Pooled HR-effect size (95%CI)*** | **I^2^** | **P Value Hetero-geneity** | **Certainty (overall score)** |
| --- | --- | --- | --- | --- | --- | --- | --- | --- | --- | --- | --- | --- |
| **Dose-response relationship between milk and CVD Mortality** | | | | | | | | | | | | |
| 7 | Observational studies | Moderate | Not serious | Not serious | Not serious | none | 10255 | 341967 | 0.87(0.79,0.96) | 36.40% | 0.151 | Moderate |
| **Dose-response relationship between milk and All-cause Mortality** | | | | | | | | | | | | |
| 7 | Observational studies | Moderate | Not serious | Not serious | Serious | none | 20712 | 350034 | 0.98 (0.93, 1.02) | 0.00% | 0.54 | Very Low |
| **Dose-response relationship between total dairy and CVD Mortality** | | | | | | | | | | | | |
| 12 | Observational studies | Moderate | Serious | Not serious | Not serious | none | 18929 | 484671 | 0.94 (0.90, 0.98) | 57.50% | 0.003 | Very Low |
| **Dose-response relationship between total dairy and All-cause Mortality** | | | | | | | | | | | | |
| 12 | Observational studies | Moderate | Serious | Not serious | Not serious | none | 76959 | 525897 | 0.97(0.94,<1.00) | 70.90% | < 0.001 | Very Low |
| **Dose-response relationship between cheese and CVD Mortality** | | | | | | | | | | | | |
| 8 | Observational studies | Moderate | Not serious | Not serious | Not serious | none | 16069 | 394567 | 0.95 (0.90, 0.99) | 0.00% | 0.578 | Moderate |
| **Dose-response relationship between cheese and All-cause Mortality** | | | | | | | | | | | | |
| 10 | Observational studies | Moderate | Not serious | Not serious | Serious | none | 69239 | 541609 | 0.99(0.97, >1.00) | 24.10% | 0.2 | Very Low |
| **Dose-response relationship between yogurt and CVD Mortality** | | | | | | | | | | | | |
| 9 | Observational studies | Moderate | Not serious | Not serious | Not serious | none | 19214 | 500601 | 0.89 (0.83, 0.95) | 0.00% | 0.588 | Moderate |
| **Dose-response relationship between yogurt and All-cause Mortality** | | | | | | | | | | | | |
| 10 | Observational studies | Moderate | Not serious | Not serious | Not serious | none | 61233 | 505127 | 0.89 (0.83, 0.96) | 31.30% | 0.133 | Moderate |

* Pooled effect size for typical serving doses of Cheese (15 g) and effect sizes for 200-gram doses of total dairy, milk, yogurt.

Data deriving from cohort studies begin with a high grading (due to use of ROBINS-E)

**6 Supplementary Figure 2 A-H Sensitivity Analysis Plot**

**
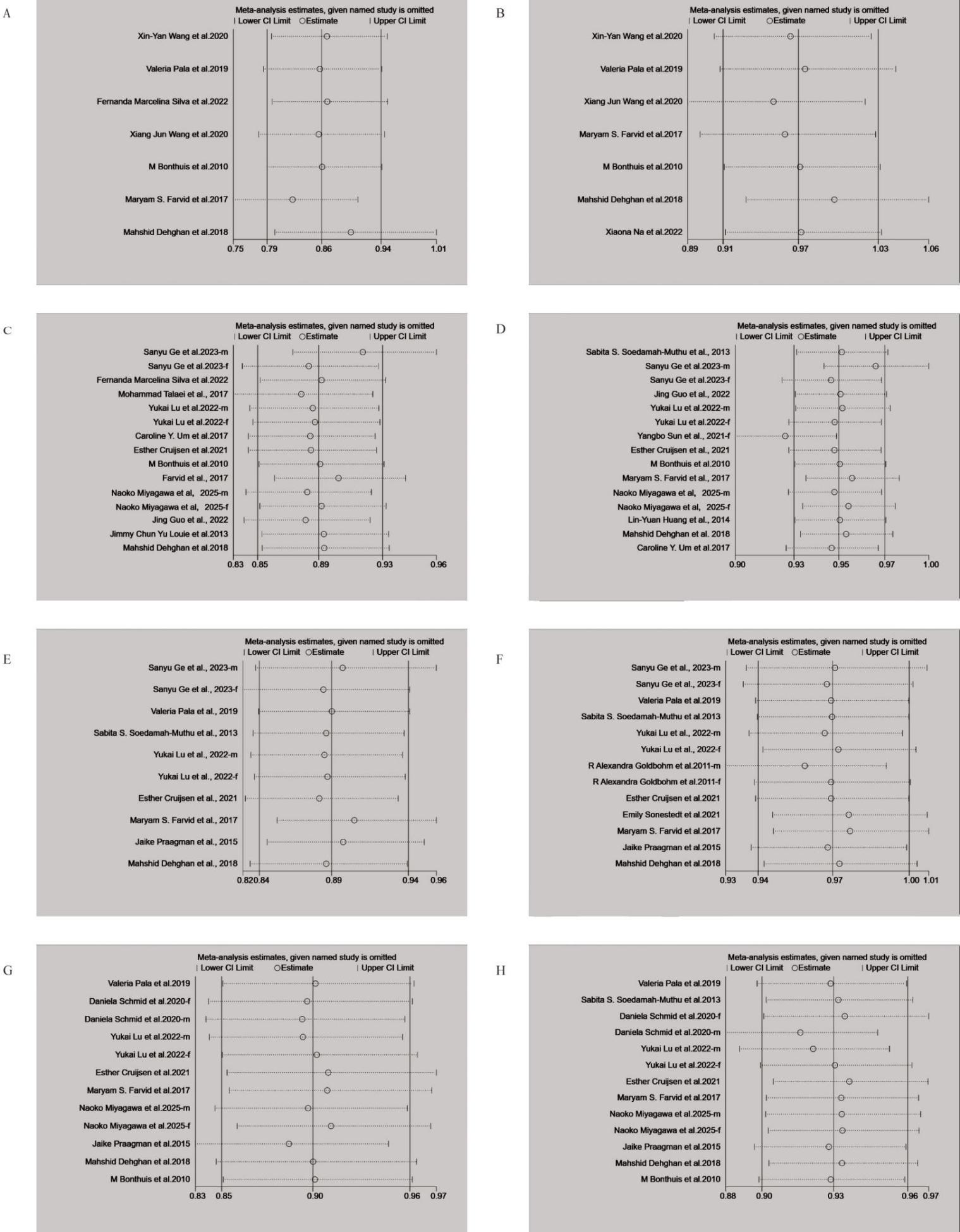
**

**7 Supplementary Figure 3 Sensitivity Analysis Plot (Total Dairy Intake vs. CVD Mortality: Including the Study by Lin-Yuan Huang et al., 2014)**

**
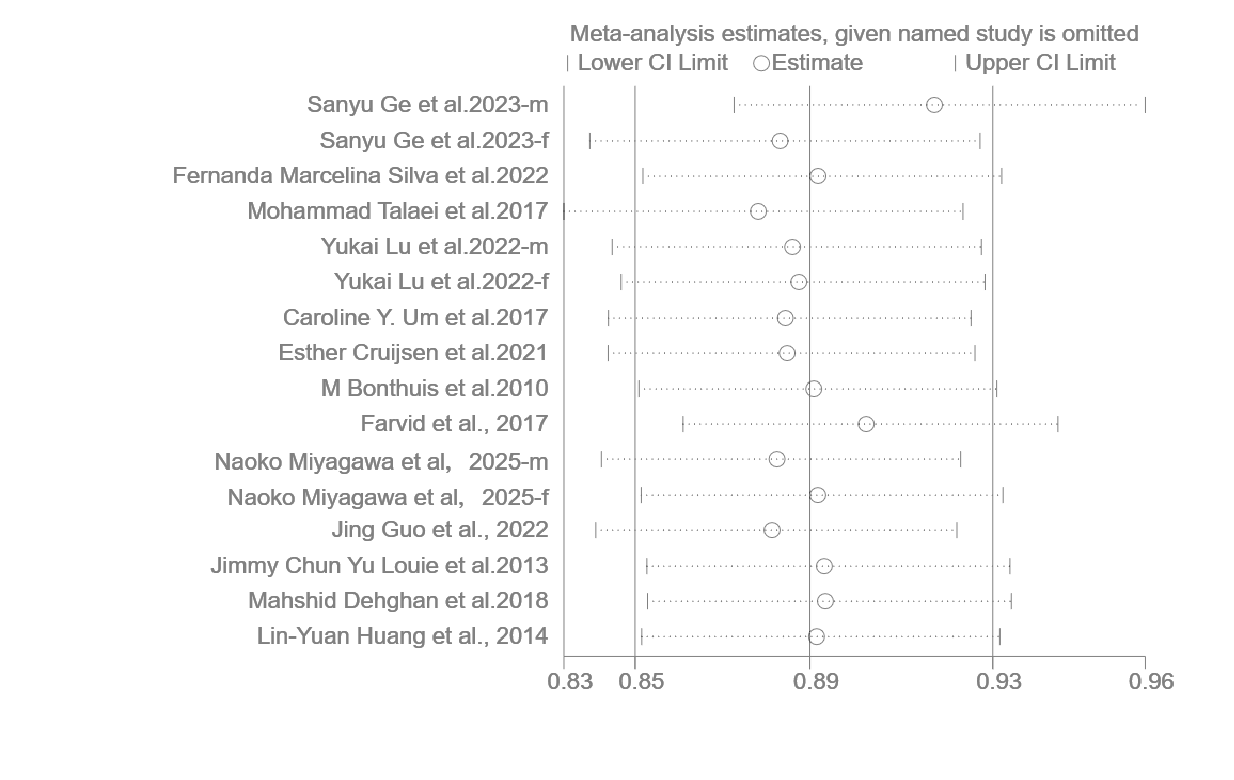
**

**9 Supplementary Table 5 Results of meta-regression**

To explore the sources of heterogeneity across studies and investigate the dose-response relationships between milk and cardiovascular disease (CVD) mortality, as well as between total dairy and CVD mortality, univariate and multivariate meta-regression analyses were performed separately in this study: the association analysis of milk and CVD mortality included 15 analysis units (consisting of 7 non-sex-stratified studies and 8 subgroups from 4 sex-stratified studies), while the association analysis of total dairy and CVD mortality included 16 analysis units (consisting of 10 non-sex-stratified studies and 6 subgroups from 3 sex-stratified studies). All models were weighted by the within-study standard error (wsse(se)), with hazard ratio (HR) values presented using exponentiated coefficients (eform); the Knapp-Hartung modification was applied to improve the robustness of small-sample data, and the between-study variance was estimated by the restricted maximum likelihood (REML) method. The key results are presented in Supplementary Tables 5.1-5.4.

**Supplementary Table 5.1 Results of univariate meta-regression analyses for milk-CVD mortality association**

| **Variables** | **HR（exp(β)）** | **Std. Err.** | **t** | **P** | **95%CI** | **Adj R²（%）** | **Residual I²**  **(%)** |
| --- | --- | --- | --- | --- | --- | --- | --- |
| Sex | 1.1910 | 0.1000 | 2.08 | 0.058 | 0.9934-1.4279 | 19.69 | 90.02 |
| Region | 1.1299 | 0.1861 | 0.74 | 0.472 | 0.7916-1.6127 | 3.23 | 89.29 |
| Total number | 1.8482 | 0.6856 | 1.66 | 0.122 | 0.8293-4.1190 | 6.04 | 92.01 |
| Median Follow up Years | 1.3540 | 0.2438 | 1.68 | 0.116 | 0.9177-1.9977 | 1.09 | 91.48 |
| Note: 1. P<0.10 indicated marginally significant, and P<0.05 indicated statistically significant; 2. All models were adjusted with Knapp-Hartung modification.3. 15 analysis units included: 7 non-sex-stratified studies and 8 subgroups from 4 sex-stratified studies. | | | | | | | |

**Supplementary Table 5.2 Results of multivariate meta-regression analysis for milk-CVD mortality association (including Sex, Region, Median Follow up Years, Total number)**

| **Variables** | **HR（exp(β)）** | **Std. Err.** | **t** | **P** | **95%CI** |
| --- | --- | --- | --- | --- | --- |
| Sex | 1.1439 | 0.1102 | 1.40 | 0.193 | 0.9229-1.4178 |
| Region | 1.2934 | 0.1750 | 1.90 | 0.086 | 0.9568-1.7485 |
| Total number | 1.8602 | 0.6834 | 1.69 | 0.122 | 0.8205-4.2173 |
| Median Follow up Years | 1.1358 | 0.2172 | 0.67 | 0.520 | 0.7418-1.7393 |
| Intercept (_cons) | 0.1428 | 0.1199 | -2.32 | 0.043 | 0.0220-0.9271 |
| Model characteristics: τ²=0.04024, residual I²=85.18%, Adj R²=34.02%; joint effect: F=2.69, P=0.093. Notes: 1. Coding and significance criteria were consistent with Supplementary Table 5.1 | | | | | |

**Supplementary Table 5.3 Results of univariate meta-regression analyses for total dairy-CVD mortality association**

| **Variables** | **HR（exp(β)）** | **Std. Err.** | **t** | **P** | **95%CI** | **Adj R²（%）** | **Residual I²**  **(%)** |
| --- | --- | --- | --- | --- | --- | --- | --- |
| Sex | 1.0219 | 0.0696 | 0.32 | 0.755 | 0.8829-1.1827 | -26.85 | 64.83 |
| Region | 1.0379 | 0.1191 | 0.32 | 0.751 | 0.8114-1.3274 | -8.28 | 63.65 |
| Total number | 0.9342 | 0.1344 | -0.47 | 0.643 | 0.6861-1.2719 | 6.18 | 62.95 |
| Median Follow up Years | 1.1378 | 0.1428 | 1.03 | 0.321 | 0.8692-1.4893 | 6.34 | 62.18 |
| Notes: 1. P<0.10 indicated marginally significant, and P<0.05 indicated statistically significant; 2. All models were adjusted with Knapp-Hartung modification; 3. 16 analysis units included: 10 non-sex-stratified studies and 6 subgroups from 3 sex-stratified studies. | | | | | | | |

**Supplementary Table 5.4 Results of multivariate meta-regression analysis for total dairy-CVD mortality association(including Sex, Region, Median Follow up Years, Total number)**

| **Variables** | **HR（exp(β)）** | **Std. Err.** | **t** | **P** | **95%CI** |
| --- | --- | --- | --- | --- | --- |
| Sex | 1.0170 | 0.1069 | 0.16 | 0.875 | 0.8069-1.2819 |
| Region | 1.1432 | 0.2319 | 0.66 | 0.523 | 0.7315-1.7866 |
| Total number | 1.0873 | 0.2653 | 0.34 | 0.738 | 0.6355-1.8604 |
| Median Follow up Years | 1.1970 | 0.2350 | 0.92 | 0.379 | 0.7771-1.8438 |
| Intercept (_cons) | 0.4564 | 0.3960 | -0.90 | 0.385 | 0.0676-3.0816 |
| Model characteristics: τ²=0.01752, residual I²=67.47%, Adj R²=-62.28%; joint effect: F=0.31, P=0.8660. Notes: 1. Coding and significance criteria were consistent with Supplementary Table 5.3. | | | | | |

**Notes:** Stratification factors: Sex(non-sex-stratified group、female、male); Region (Asia vs. Europe and America); Total sample size (<10,000 vs. ≥10,000); Follow-up duration (<10 years vs. ≥10 years).

**9 Supplementary Figure 4 Forest Plot of Subgroup Analyses for Milk and Total Dairy Intake in Association with Cardiovascular Disease (CVD) Mortality**

**
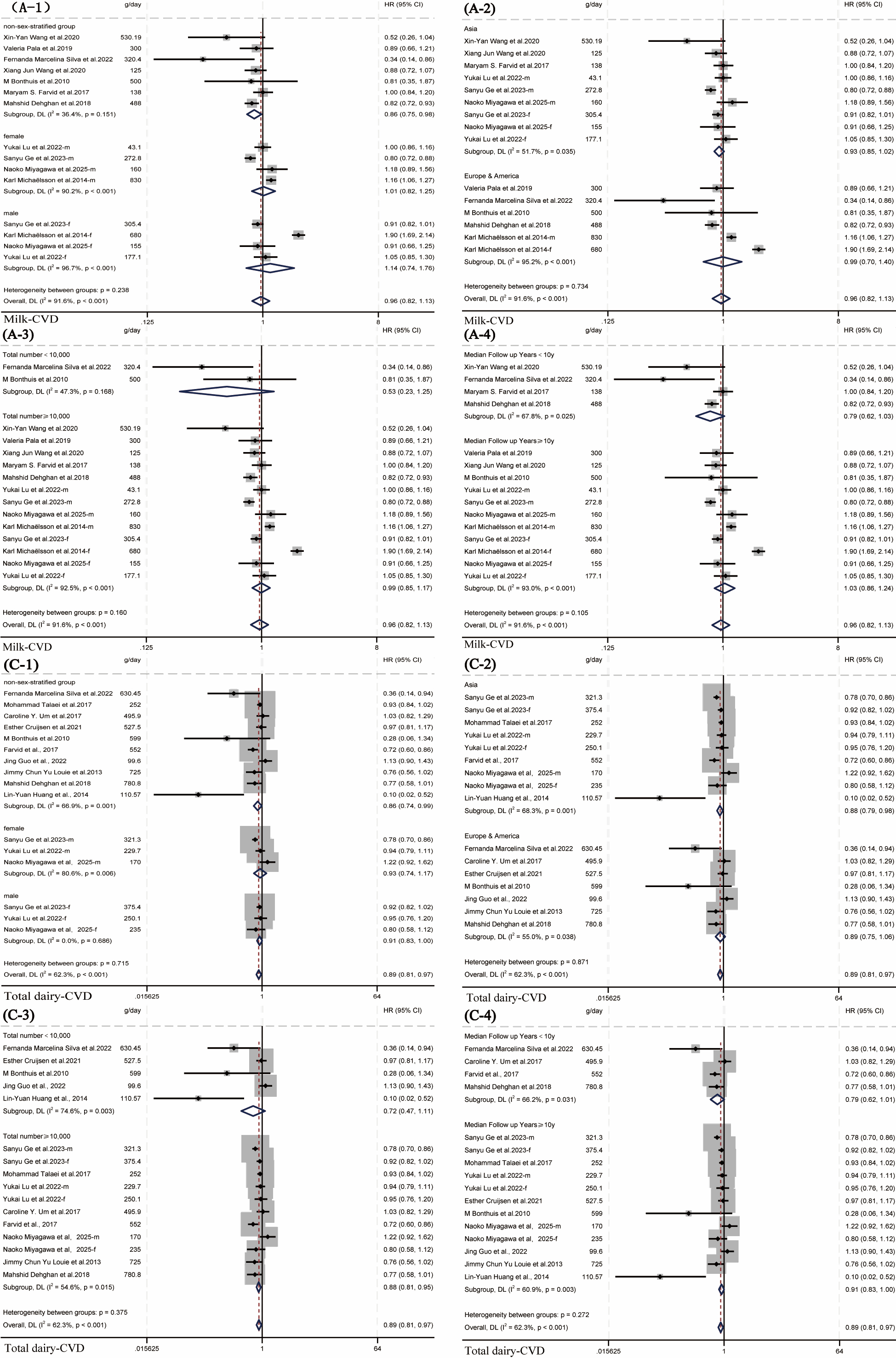
**
